# HRS–WASH axis governs actin-mediated endosomal recycling and cell invasion

**DOI:** 10.1083/jcb.201710051

**Published:** 2018-07-02

**Authors:** Ewan MacDonald, Louise Brown, Arnaud Selvais, Han Liu, Thomas Waring, Daniel Newman, Jessica Bithell, Douglas Grimes, Sylvie Urbé, Michael J. Clague, Tobias Zech

**Affiliations:** 1 Institute of Translational Medicine, Cellular and Molecular Physiology, University of Liverpool, Liverpool, England, UK; 2 Institute of Cancer Stem Cell, Dalian Medical University, Dalian, China

## Abstract

Transmembrane proteins in the sorting endosome are either recycled to their point of origin or destined for lysosomal degradation. Lysosomal sorting is mediated by interaction of ubiquitylated transmembrane proteins with the endosomal sorting complex required for transport (ESCRT) machinery. In this study, we uncover an alternative role for the ESCRT-0 component hepatocyte growth factor–regulated tyrosine kinase substrate (HRS) in promoting the constitutive recycling of transmembrane proteins. We find that endosomal localization of the actin nucleating factor Wiscott-Aldrich syndrome protein and SCAR homologue (WASH) requires HRS, which occupies adjacent endosomal subdomains. Depletion of HRS results in defective constitutive recycling of epidermal growth factor receptor and the matrix metalloproteinase MT1–MMP, leading to their accumulation in internal compartments. We show that direct interactions with endosomal actin are required for efficient recycling and use a model system of chimeric transferrin receptor trafficking to show that an actin-binding motif can counteract an ubiquitin signal for lysosomal sorting. Directed receptor recycling is used by cancer cells to achieve invasive migration. Accordingly, abrogating HRS- and actin-dependent MT1-MMP recycling results in defective matrix degradation and invasion of triple-negative breast cancer cells.

## Abstract (precis)

ESCRT-0 component HRS and actin polymerization factor WASH reside in adjacent endosomal domains. MacDonald et al. show that HRS controls WASH localization and recycling of WASH-dependent transmembrane cargo. Cargo binding to endosomal actin thus acts as sorting signal to oppose ubiquitin-mediated degradation.

## Introduction

Cell surface proteins that enter endosomes may be recycled to the plasma membrane or otherwise actively sorted toward the lysosomal pathway. The latter pathway has been well characterized in the case of ubiquitylated receptors, which engage with components of the endosomal sorting complex required for transport (ESCRT) machinery (Williams and Urbé, 2007; Henne et al., 2011). The ESCRT-0 complex, comprising hepatocyte growth factor–regulated tyrosine kinase substrate (HRS) and signal-transducing adapter molecule (STAM), provides multiple ubiquitin (Ub) interaction surfaces as well as recruiting the ESCRT-I complex via interactions between HRS and TSG101 (Bache et al., 2003; Clague and Urbé, 2003; Pornillos et al., 2003). HRS is recruited to endosomes via its Fab1, YOTB, Vac1, and EEA1 (FYVE) domain, which interacts with locally generated PtdIns3*P* (Urbé et al., 2000). The sorting endosome is subcompartmentalized into tubular and vacuolar aspects and shows segregation of proteins to specific domains within the same limiting membrane (Luini et al., 2005).

Ub is an established signal for sorting into the multivesicular body (MVB), a structure that forms upon endosome maturation. Several motifs have also been established to promote receptor endocytosis (Lauwers et al., 2009). However, no unifying intrinsic sequence has been found that affects recycling from endosomes (Jing et al., 1990; Apodaca et al., 1994; Gruenberg, 2001). The pathway has to accommodate bountiful and highly dynamic shuttling receptors for internalized intracellular nutrients such as the transferrin (Trf) receptor (TrfR) and also must provide an escape route for receptors and other plasma membrane components that have not been marked for degradation. The prevailing early view was that it largely represents a bulk-flow process (Mayor et al., 1993). Recent work has suggested that the Wiscott-Aldrich syndrome protein and SCAR homologue (WASH) complex in association with defined retromer complexes mediates the recycling of specific plasma membrane proteins (Steinberg et al., 2013). A more complex feature of the recycling pathway is represented by its ability to distribute to different regions of the cell, for example the leading edge of migrating cells or one or the other membrane of polarized cells (Matter and Mellman, 1994). Such recycling of membrane type 1–matrix metalloproteinase (MT1-MMP) and EGF receptor (EGFR) drives cancer cell invasion (Caswell et al., 2008; Steffen et al., 2008).

The WASH complex is an endosomal Arp2/3 activator that stimulates the polymerization of F-actin (Derivery et al., 2009; Gomez and Billadeau, 2009; Nagel et al., 2017). It facilitates retrograde trafficking from endosomes to Golgi (cation-independent mannose-6-phosphate receptor [ci-M6PR]; Gomez and Billadeau, 2009) and recycling from endosomes to the plasma membrane (α5β1 integrin [Zech et al., 2011]; low-density lipoprotein receptor [Bartuzi et al., 2016]). Currently, the mechanisms of WASH complex recruitment and activation are only partially understood. An interaction between FAM21 and the retromer component VPS35 was shown to be important for the recruitment of the WASH complex onto endosomes, and phospholipid binding may play a role in its membrane targeting (Jia et al., 2010; Harbour et al., 2012; Helfer et al., 2013). Interestingly, WASH activity can be controlled through reversible ubiquitylation, which stabilizes the WASH complex in its active form (Hao et al., 2013, 2015). Depletion of WASH has been reported to result in elongated tubules emanating from the endosome, and as such, WASH is thought to participate in membrane fission through an interaction with dynamin (Derivery et al., 2009). F-actin is required for the stabilization of tubules that are used for sorting of receptors, and it has been proposed that direct and indirect interactions between transmembrane proteins and actin sequesters receptors for recycling (Puthenveedu et al., 2010; Carnell et al., 2011; Zech et al., 2012). In this study, we took advantage of two known actin binding domains in the EGFR receptor (den Hartigh et al., 1992) and the metalloproteinase MT1-MMP (Yu et al., 2012) to investigate their influence on retrograde trafficking. We provide evidence that WASH-mediated receptor recycling and ESCRT-driven degradation at the endosome are functionally coupled by virtue of a shared requirement for the ESCRT-0 component HRS. WASH localization to the endosome is dependent on HRS, whereas the two systems compete for the sorting of receptors into respective subdomains. We identify the function of the intrinsic actin-binding domains (ABDs) of EGFR and MT1-MMP as directing the default sorting to recycling under steady-state conditions and show that HRS-orchestrated endosomal actin is required for MT1-MMP–dependent invasive cell migration of breast cancer cells.

## Results

### HRS occupies a separate subdomain but is required for the recruitment of WASH to endosomes

**Figure 1.**
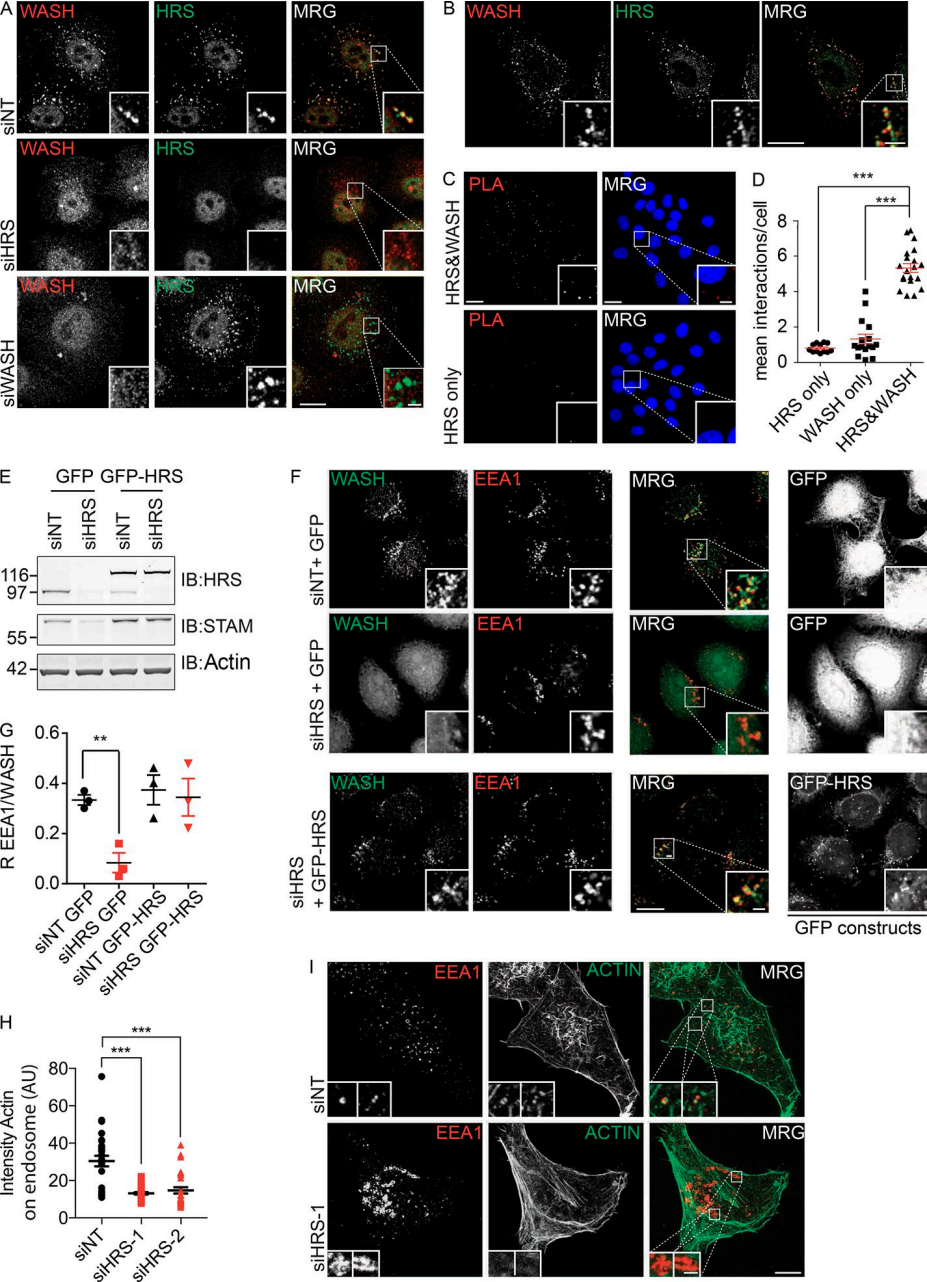
**HRS is required for the endosomal recruitment of WASH. (A)** HeLa cells were treated with the indicated siRNA over 120 h before fixation in 4% PFA/PBS and labeling with antibodies targeting HRS and WASH. Maximum-projection images. **(B)** Airyscan single confocal slice images of HeLa cells fixed and labeled for WASH and HRS. **(C and D)** PLA of HeLa cells probed for both HRS and WASH or technical single WASH or HRS antibody controls. Data represented as mean number of signals per cell. Maximum-projection images (nuclei stained with DAPI). Bars, 20 µm. **(E and F)** HeLa S3 Flp-In cells stably transfected with GFP-HRS (mouse) were treated with siRNA targeting endogenous HRS over 120 h before fixation in 4% PFA/PBS or lysis in NP-40 buffer. Single confocal slice images. Molecular masses are given in kilodaltons. IB, immunoblot. **(G)** Quantification of Pearson’s R correlation between EEA1 and WASH (>150 cells total). **(H and I)** HeLa cells were treated with siRNA over 120 h before fixation in 4% PFA/PBS and labeling with antibodies targeting EEA1 and 647-phalloidin. Quantification of sum intensity of actin on endosome. Maximum-projection images (>30 cells total). *n* = 3; error bars indicate SEM. **, P < 0.01; ***, P < 0.001. Statistical analysis, one-way ANOVA, Dunnett’s post hoc test. Bars: (main images) 10 µm; (insets) 2 µm. MRG, merge.

Costaining of fixed cells with WASH and HRS antibodies revealed a high degree of colocalization in HeLa and MDA–MB-231 cells (Figs. 1 A and S1 A). We further used a HeLa Flp-In cell line stably expressing GFP-tagged mouse HRS (GFP-mHRS) at near endogenous levels, to transiently express mCherry-mWASH. Again we observed a high degree of overlap between the two proteins and their comigration on dynamic endosomes (Video 1). Nevertheless, using Airyscan-based superresolution microscopy to image at 100-nm resolution, we could resolve spatial differences representing their concentration in adjacent subdomains of the endosome (Fig. 1 B). To confirm this, we used overexpression of a constitutively active form of Rab5 (Rab5Q79L), which induces the formation of large endosomes by promoting homotypic fusion (Barbieri et al., 1996). Costaining these cells for WASH and HRS, we observed a separation of WASH and HRS domains on the limiting membrane (Fig. S1 B). To investigate how closely juxtaposed these endosomal subdomains are, we used a proximity ligation assay (PLA) that showed that HRS and WASH can be found within a 40-nm distance from each other, the maximum working distance of this assay (Fig. 1, C and D; and Fig. S1 C). These orthogonal experiments demonstrate that WASH and HRS are localized to the same endosome in HeLa cells but are separated out into adjacent subdomains on the limiting membrane.

We next sought to determine whether there was a functional relationship between HRS and WASH. We depleted either HRS or WASH using siRNA. Although WASH depletion did not interfere with HRS localization, depletion of HRS resulted in the loss of WASH staining at endosomes in both HeLa and MDA–MB-231 cells (Fig. 1, A, F, and G; and Fig. S1, A, D, and E). Using EEA1 as an endosomal marker in HRS-depleted cells, we saw a concomitant loss of endosomal F-actin and Arp2/3 complex that is a predicted consequence of the mislocalization of WASH (Fig. 1, H and I; and Fig. S1, F and G).

**Figure 2.**
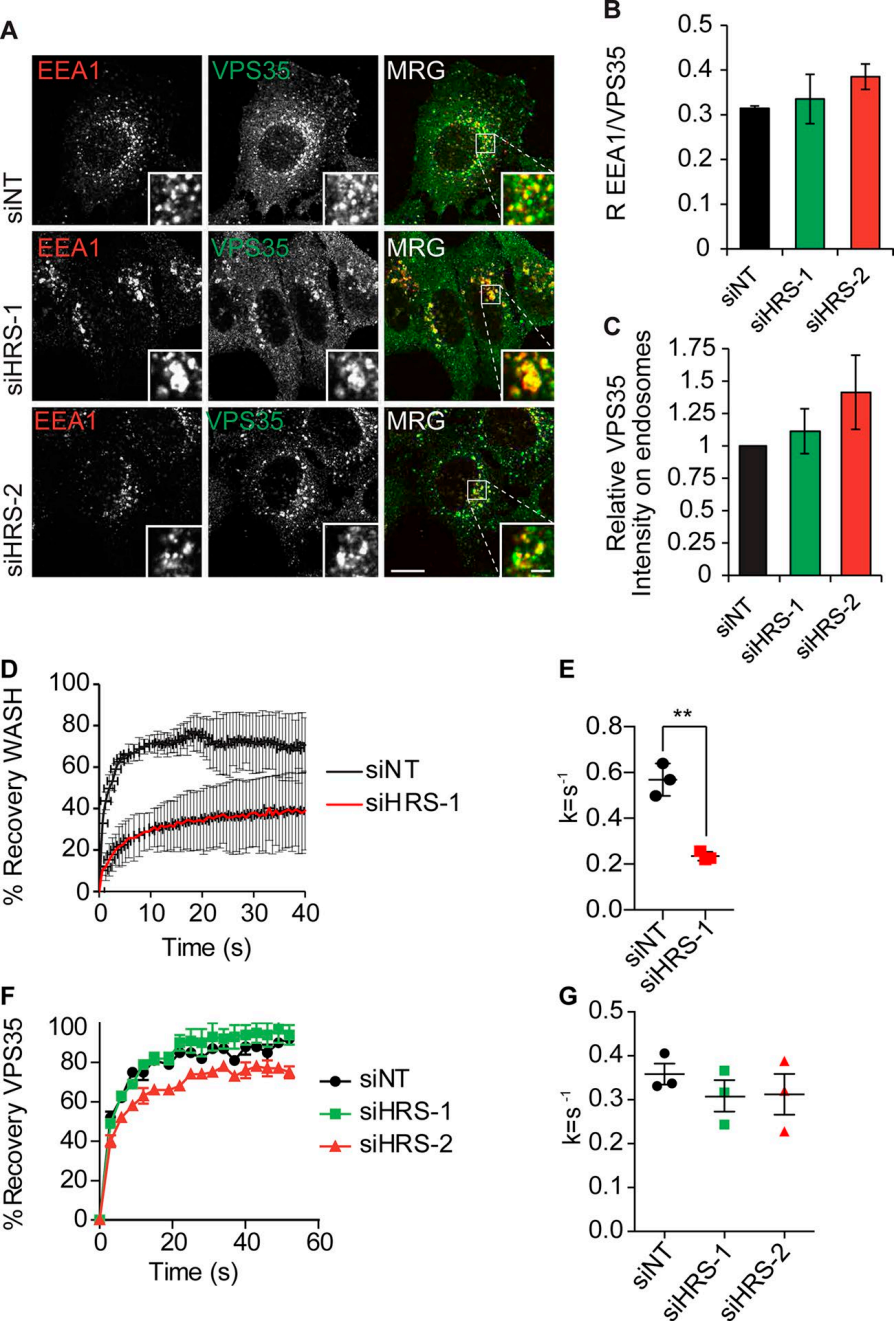
**HRS is required for the endosomal recruitment of WASH independent of VPS35. (A)** HeLa cells were treated with the indicated siRNA over 120 h before fixation in 4% PFA/PBS and labeling with antibodies targeting VPS35 and EEA1. Single confocal slice images. Bars: (main images) 10 µm; (insets) 2 µm. MRG, merge. **(B)** Pearson’s R correlation value between VPS35 and EEA1 (10 images per condition, >150 cells total). **(C)** Relative intensity of VPS35 on EEA1-positive endosomes normalized to endosome size (>150 cells total). **(D)** HeLa cells were depleted for HRS over 120 h before transfection with mCherry-WASH construct 24 h before imaging; the cells were subjected to photobleaching with a 594-nm laser. Fluorescence recovery was measured using the FRAP tool in the Slidebook image analysis suite. **(E)** The rate constants (k = s^−1^) for the recovery curves were extracted using Prism. The mean rate constants of three independent experiments are plotted (>10 cells per condition per experiment). **, P < 0.01. **(F)** HeLa cells were depleted of HRS with siRNA over 120 h before transfection with YFP-VPS35 construct 24 h before imaging. Cells were incubated with 647-dextran for 15 min before imaging to mark the endocytic network, and YFP-VPS35–positive endosomes were subjected to photobleaching with 594-nm laser light. **(G)** The rate constants (k = s^−1^) for the recovery curves were extracted using GraphPad Prism. The mean rate constants of three independent experiments are plotted (>10 cells per condition per experiment). *n* = 3; error bars indicate SEM.

Immunoblot analysis of HRS-depleted HeLa cells revealed that there was a similar reduction in WASH (percentage of small interfering nontargeting control [NT; siNT], siHRS-1, 71 ± 16%; siHRS-2, 58 ± 3%) and the retromer component VPS35 (percentage of siNT, siHRS-1, 60 ± 6%; siHRS-2, 46 ± 3%) protein levels in HeLa cells, but in MDA–MB-231 cells we observed no significant changes in the levels of WASH, despite the observed loss of WASH staining from the endosome (Fig. S2, A–F). To confirm that the loss of WASH from endosomes was caused by the depletion of HRS, we performed parallel experiments in isogenic Flp-In cell lines expressing either GFP or GFP-mHRS (Fig. 1 E). In control GFP-expressing cells, the early endosomal marker EEA1 and WASH colocalized (Pearson’s R coefficient [R], 0.33 ± 0.02), but after endogenous HRS depletion with siRNA targeting human HRS, this was reduced to background levels (R, 0.08 ± 0.055). However, in the cell lines expressing siRNA-resistant GFP-mHRS, there were no measurable differences in the colocalization of WASH with EEA1 (GFP-mHRS + siHRS R, 0.34 ± 0.1) after treatment with the same siRNA oligonucleotides (Fig. 1, F and G). We observed no loss in the endosomal pool of VPS35 or the COMMD–CCDC22–CCDC93 (CCC) complex member COMMD1 that is also linked to the WASH complex (Fig. 2, A–C; and Fig. S1, H and I; Phillips-Krawczak et al., 2015; Bartuzi et al., 2016). This confirms that HRS expression is specifically required for correct localization and function of WASH.

To test whether the dynamics of WASH recruitment onto the endosomal membrane are governed by HRS, we performed FRAP experiments using mCherry-WASH (Fig. 2 D). HeLa cells were depleted of HRS using siRNA and then transfected with GFP-EEA1 and mCherry-WASH. WASH and EEA1 positive endosomes were subjected to photobleaching, and the recovery of mCherry-WASH was measured. There was a significant decrease in the rate of recovery after HRS depletion (siNT k, 0.504 ± 0.071; siHRS k, 0.171 ± 0.046 [k = s^−1^]; *t* test P < 0.01; Fig. 2 E). In contrast, there was no observable change in the recruitment dynamics of VPS35 after HRS depletion (Fig. 2, F and G). Collectively, the data demonstrate that HRS is required for the correct recruitment and localization of WASH to endosomes.

We sought to identify domains in HRS responsible for the recruitment of WASH to endosomes. We tested several HRS domain deletion constructs, known to retain endosomal localization, and found that expression of the combined VHS-FYVE domain alone was sufficient to rescue the endosomal localization of WASH (Fig. S3, A–C). The FYVE domain is necessary for the recruitment of HRS onto endosomes in a PtdIns3*P*-dependent manner (Urbé et al., 2000), whereas the VHS domain is thought to act as an interaction module. In GGA proteins, the VHS domain can directly bind to cargo to facilitate retrograde transport (Mao et al., 2000; Puertollano et al., 2001). We tested whether HRS-mediated WASH recruitment is sensitive to cargo accumulation at the endosome. We used 100 µM primaquine to block recycling of transmembrane proteins in HeLa cells and observed that this led to accumulation of GFP-VHS-FYVE, EGFR, and WASH, but not the PtdIns3*P* sensor GFP-FENS-FYVE, on the endosomal membrane (Fig. S3, D–F; and not depicted).

### HRS is required for the actin-mediated recycling of WASH-dependent cargo

The preceding results suggested that HRS may be able to govern endosomal recycling of specific receptors through recruitment of WASH and localized control of actin dynamics. To check this, we initially monitored the distribution of ci-M6PR, a transmembrane protein that shuttles between the TGN and endosomes. At steady-state in control HeLa cells, ci-M6PR was localized to the TGN. After depletion of HRS or WASH, ci-M6PR redistributed to EEA1-positive endosomes away from TGN46 (Fig. S4, A–D). In the absence of ci-M6PR recycling from the endosome back to the TGN, the receptor cannot engage with any newly synthesized acid hydrolases such as cathepsin D, which are therefore missorted at the TGN and secreted into the extracellular environment (Lobel et al., 1989). We precipitated proteins from conditioned media of either control or HRS-depleted HeLa cells. In HRS-depleted cells, we observed an increase in the levels of immature cathepsin D in the medium, providing biochemical evidence for a defect in ci-M6PR shuttling (Fig. S4 E).

**Figure 3.**
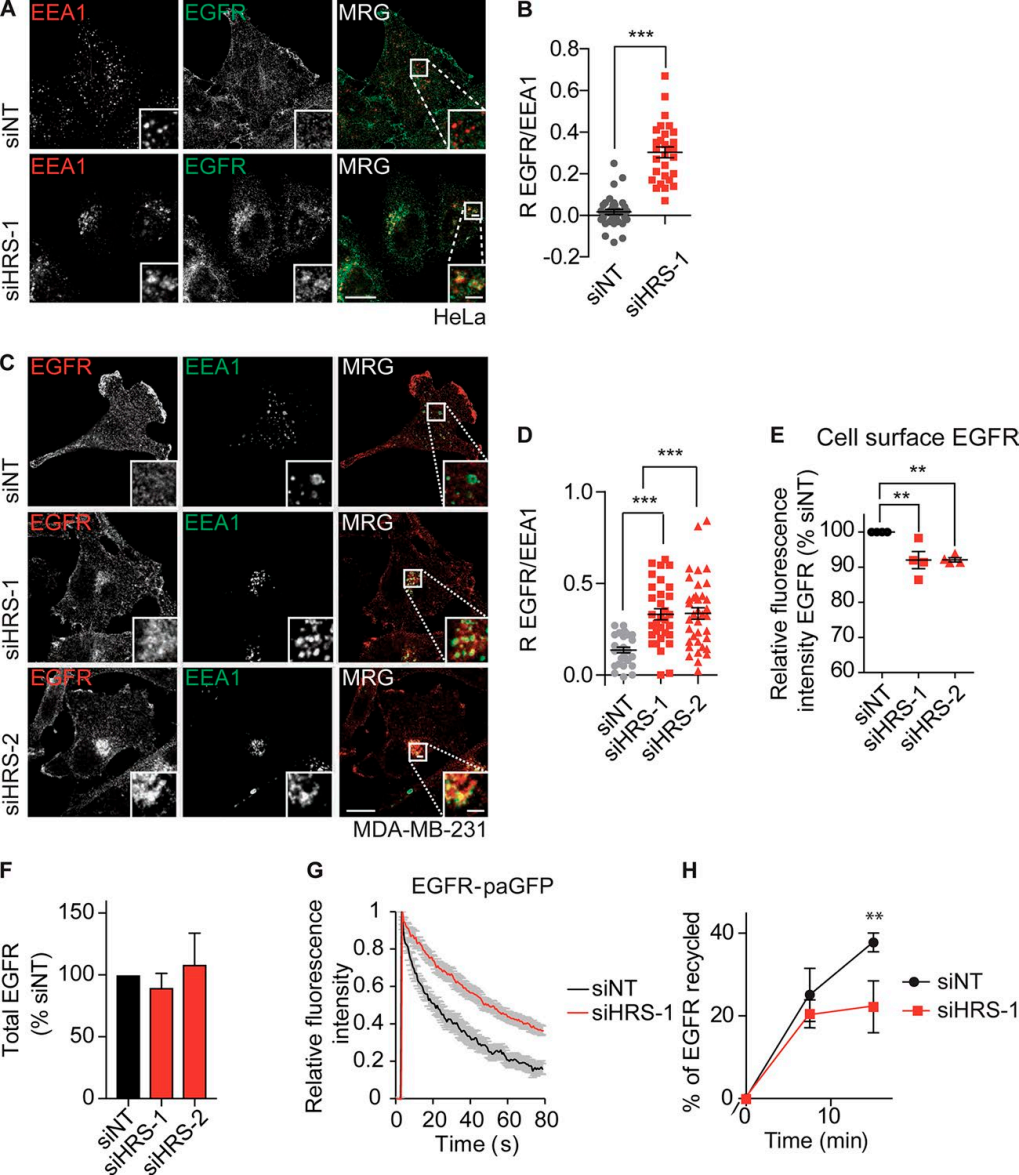
**HRS is required for receptor recycling. (A)** HeLa cells were treated with siRNA for 120 h before fixation in 4% PFA/PBS and labeling with antibodies targeting EEA1 and EGFR. **(B)** Pearson’s R correlation value between EGFR and EEA1. Single confocal slice images. **(C)** MDA–MB-231 cells were depleted for 120 h with siRNA before fixation in 4% PFA/PBS and labeling with antibodies targeting EEA1 and EGFR. Single-slice images. Bars: (main images) 10 µm; (insets) 2 µm. MRG, merge. **(D)** Pearson’s R correlation value between EGFR and EEA1. **(B–D)** *n* = 3; all data points plotted; >10 images per condition; >150 cells total. **(E)** Cell surface levels of EGFR were measured by flow cytometry. The mean fluorescent intensity was plotted as a percentage of the siNT control for each individual experiment (*n* = 4). **(F)** Total EGFR levels in cell lysates normalized to siNT (*n* = 3). **(G)** siHRS- and control-depleted HeLa cells were transfected with EGFR-paGFP fusion constructs and mCherry-RAB4. Trafficking from the endosome was measured by quantifying the decrease in GFP fluorescence in the endosome normalized to rate of photobleaching (*n* = 9 over two independent experiments). **(H)** MDA–MB-231 cells were treated with the indicated siRNA for 120 h before being surface-labeled with N-hydroxysuccinimide–SS-biotin on ice and subsequently warmed to generate an internal pool. Then, surface biotin was stripped. The cells were warmed for 7.5 min or 15 min to allow for recycling. The cell surface was stripped again to determine the percentage of recycled receptor compared with total internal pool (*n* = 3). Error bars indicate SEM. **, P < 0.01; ***, P < 0.001. Statistical analysis, one-way ANOVA with Dunnett’s post hoc test, except B and H are *t* tests.

We next tested whether HRS depletion had an effect on the steady-state trafficking of endogenous EGFR, which is also known to accumulate in endosomes after loss of WASH (Gomez et al., 2012). Under steady-state conditions, there was an accumulation of EGFR in an EEA1-positive endosomal compartment but not the TGN in HeLa (Fig. 3, A and B; and Fig. S4 F) and MDA–MB-231 cells (Fig. 3, C and D). We observed a concomitant loss of cell surface EGFR levels (Fig. 3 E) but no significant change in total EGFR levels after HRS depletion (Fig. 3 F). To ascertain that the accumulation of EGFR was a result of defective retrograde traffic under our experimental conditions, we blocked endosomal receptor recycling with primaquine and observed a comparable accumulation of EGFR in endosomes (Fig. S3 F). We used photoactivatable (pa) GFP coupled to EGFR (EGFR-paGFP) to determine whether this EGFR accumulation was caused by a failure to recycle EGFR out of endosomes and to directly measure the residence time of the receptor in Rab4 positive endosomes in HeLa cells. After HRS depletion, there was a significant decrease in the rate of EGFR exit from the endosome compared with control cells (siNT k, 0.0462 ± 0.0168; siHRS k, 0.0215 ± 0.011 [k = s^−1^]; *t* test P < 0.01; Fig. 3 G and Video 2). To further confirm this observation, we performed biochemical EGFR trafficking ELISA assays based on reversible biotinylation that showed a significant reduction in the percentage of receptor that was recycled (Fig. 3 H; *t* test P < 0.01).

**Figure 4.**
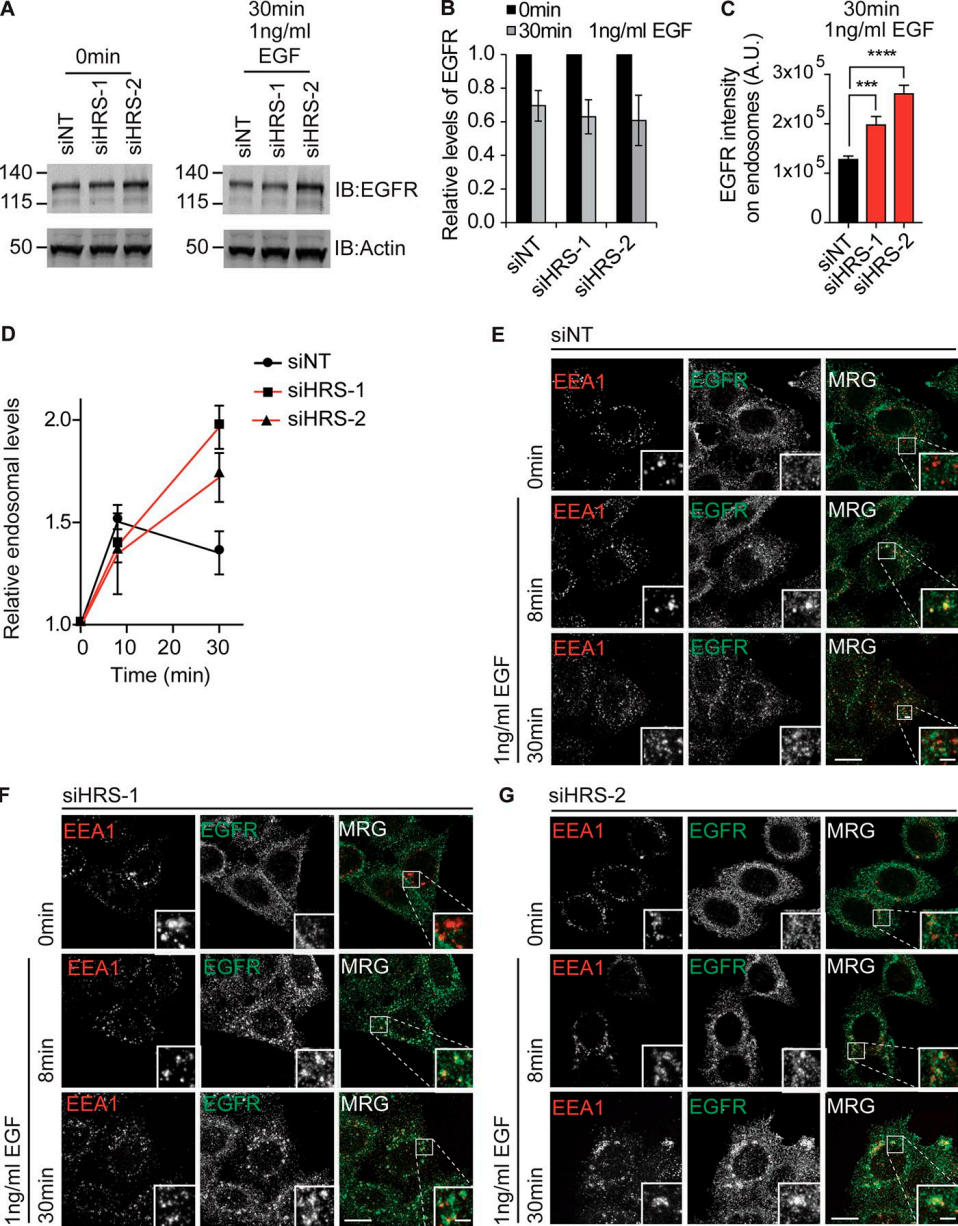
**HRS is required for activated EGFR recycling. (A)** HeLa cells were treated with the indicated siRNA over 120 h and serum-starved for 2 h before stimulation with 1 ng/ml EGF over the indicated time course and lysis in RIPA buffer. IB, immunoblot. Molecular masses are given in kilodaltons. **(B)** Quantification of degradation normalized to time 0 min. **(C and D)** Quantification of relative fluorescence intensity of EGFR in EEA1-positive endosomes. Values were normalized to endosome size. **(C)** EGFR endosomal intensity after 30-min treatment with 1 ng/ml EGF. **(D)** EGFR intensity in EEA1-positive endosomes over time course of stimulation with 1 ng/ml EGF, values were normalized to time point 0 for each condition. *n* = 3; error bars indicate SEM; approximately >75 cells total. **(E–G)** HeLa cells treated as before, followed by fixation in 4% PFA/PBS and staining with antibodies targeting EEA1 and EGFR. Sum intensity projection images. ***, P < 0.001; ****, P < 0.0001. Statistical analysis, one-way ANOVA with Dunnett’s post hoc test. Bars: (main images) 10 µm; (insets) 2 µm. MRG, merge.

To determine whether HRS had an effect on trafficking of activated EGFR, we serum-starved HeLa cells for 2 h before stimulation with 1 ng/ml EGF, an experimental setup that has previously been identified to increase the internalization rate of the receptor but not the degradation of the receptor (Sigismund et al., 2013). We did not see significant changes in the degradation rate of EGFR in HeLa cells treated with 1 ng/ml EGF upon loss of HRS compared with WT cells (Fig. 4, A and B). We did, however, observe an increase in the levels of EGFR in EEA1-positive endosomes in HRS depleted cells after 30 min compared with control cells (Fig. 4, C–G). As there was no change in the EGFR protein levels, we conclude that the change in distribution of EGFR upon HRS loss is a result of changes in the ability of the EGFR to recycle out of the EEA1/VPS35/WASH-positive endosomes.

**Figure 5.**
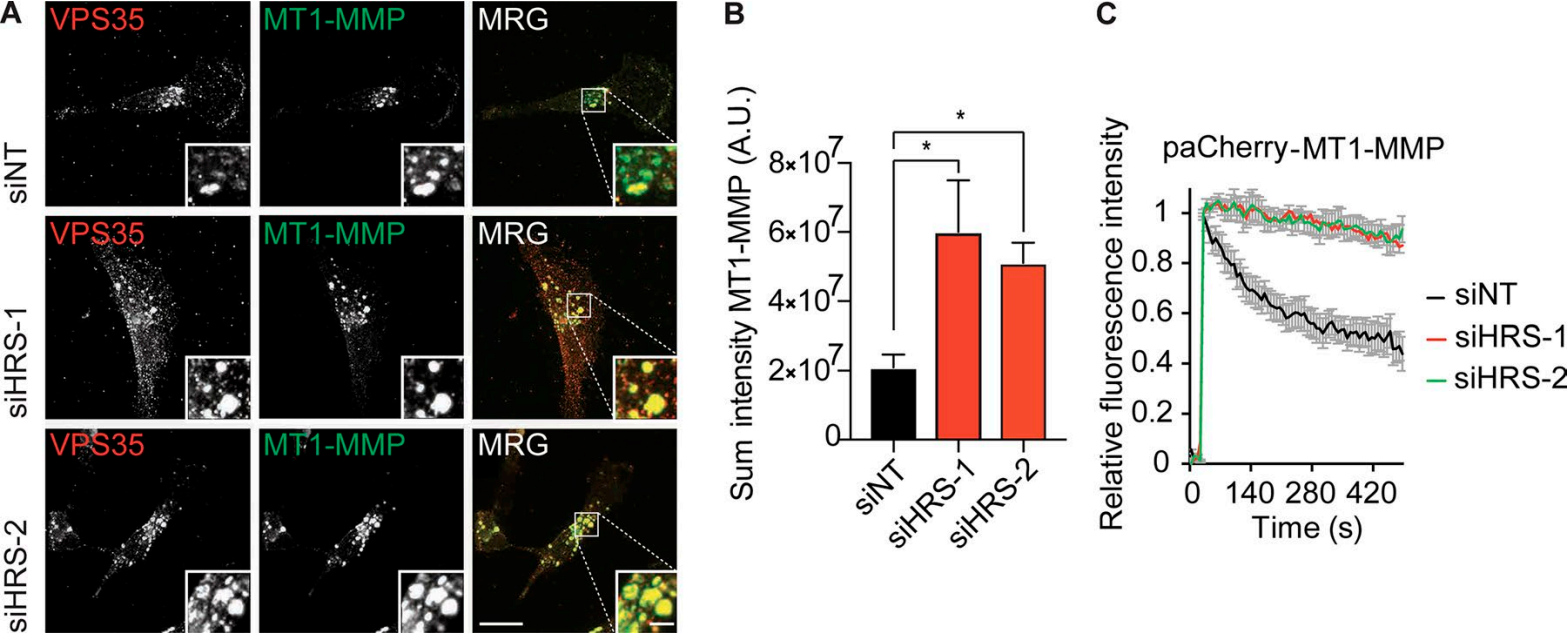
**HRS is required for MT1-MMP endosomal recycling. (A)** MDA–MB-231 cells were treated twice with siRNA over 120 h before fixation and subsequent treatment with guanidinium hydrochloride. Sum intensity projections. Bars: (main images) 10 µm; (insets) 2 µm. MRG, merge. Brightness and contrast of main images and insets was adjusted uniformly across panels to best display plasma membrane or endosomal staining, respectively. **(B)** Quantification of sum intensity of MT1-MMP in a VPS35 mask. *n* = 50 cells over three independent experiments. *, P < 0.05. Statistical analysis, one-way ANOVA, Dunnett’s post hoc test. **(C)** MDA–MB-231 cells were treated with siRNA targeting HRS for 120 h and transfected with paCherry–MT1-MMP and GFP-EEA1. Trafficking from the endosome was measured by quantifying the decrease in paCherry fluorescence in the endosome. *n* = 6 independent experiments; >20 cells total; error bars indicate SEM.

To confirm our results with another WASH complex–dependent cargo, we next looked at trafficking of the proinvasive MMP MT1-MMP in the triple-negative breast cancer cell line MDA–MB-231 (Monteiro et al., 2013). HRS-depleted and control MDA–MB-231 cells were stained for endogenous MT1-MMP. This showed a concentration of MT1-MMP in retromer-positive endosomal compartments that was greatly increased upon HRS depletion (Fig. 5, A and B; and Fig. S5 A). The dynamics of MT1-MMP recycling from endosomes were tested by transfection with a pa mCherry–MT1-MMP (paCherry–MT1-MMP) construct and GFP-EEA1. We observed that there was a significant delay in the recycling of the receptor from the endosome (Fig. 5 C). These results confirm a robust blockade in the recycling of WASH-dependent cargo upon HRS depletion.

### Direct actin binding of receptors is required for efficient endosomal sorting

**Figure 6.**
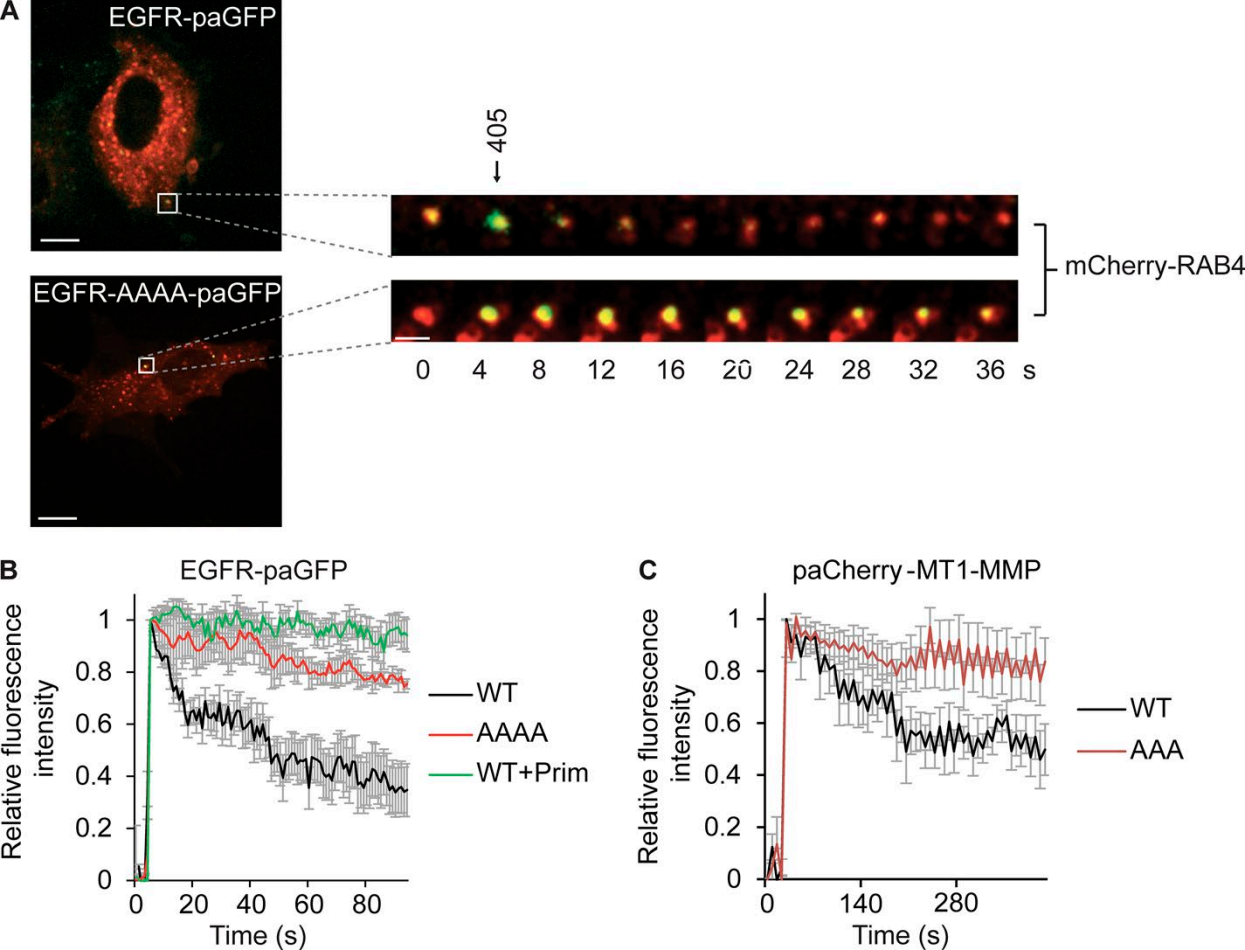
**Actin binding is required for the efficient sorting of receptors. (A–C)** HeLa cells were transfected with EGFR and EGFR actin binding mutant YLIP/AAAA (1,016–1,019) coupled to paGFP and mCherry-Rab4. GFP was activated in Rab4-positive endosomes by exposure to 405-nm laser light, and the rate of recycling was measured by quantifying the decrease in paGFP fluorescence from the endosome. Pretreatment with 100 µM primaquine (Prim) to block receptor recycling was used as a negative control. **(A)** Representative activation in Rab4-positive endosomes (taken from Video 3; a Gaussian blur has been added). Bars, 10 µm. **(B)** Representative traces from an individual experiment (>10 cells per experiment per condition; four independent experiments). **(C)** MDA–MB-231 with paCherry fusion MT1-MMP constructs WT or LLY/AAA (571–573) and GFP-EEA1. paCherry constructs were activated in GFP-EEA1–positive endosomes and quantified as above (graph mean traces over three individual experiments; >20 cells total). Error bars indicate SEM.

A loss of the HRS–WASH axis caused a decrease in endosomal F-actin and a blockade in recycling. Thus, we wondered whether proteins could directly or indirectly bind to F-actin to facilitate this transport step (Zech et al., 2012). To test this hypothesis, we used two recycling proteins that have previously been demonstrated to directly bind to actin, the EGFR (den Hartigh et al., 1992) and MT1-MMP (Yu et al., 2012). We mutated the previously identified minimal sequences required for actin binding for both EGFR and MT1-MMP, YLIP/AAAA (1,016–1,019) and LLY/AAA (571–573), respectively, to assess changes in the localization and trafficking of the receptors. To investigate actin-dependent EGFR recycling, we used photoactivation experiments from mCherry-Rab4–positive endosomes. We observed quicker sorting of the WT receptor into vesicles emanating from Rab4-positive endosomes (Fig. 6, A and B; and Video 3), whereas the actin-binding deficient receptors were excluded from the bulk of tubules (Fig. 6, A and B; and Video 3). We could also observe gross changes in the localization of our overexpressed constructs, with the YLIP/AAAA being predominantly confined to endosomes whereas the WT EGFR showed more cell surface expression (Video 4). By using primaquine as a positive control for recycling inhibition (van Weert et al., 2000), we could establish that blockade of receptor recycling causes a corresponding stabilization of the fluorescence signal in the endosome (Fig. 6 B and Video 5). There was a significant decrease in the rate of exit of the actin-binding deficient EGFR from the endosomes compared with the WT receptor (WT k, 0.049 ± 0.004; AAAA k, 0.03 ± 0.003, *t* test, P = 0.02; *n* = 4 individual experiments [k = s^−1^]). When we tested the trafficking dynamics of photoactivated paCherry–MT1-MMP constructs out of EEA1-GFP–positive endosomes, we observed an almost complete blockade in the trafficking of the ABD mutant MT1-MMP LLY/AAA constructs (Fig. 6 C and Video 6). These results show that the recycling of EGFR and MT1-MMP out of the endosome is dependent on the ability of the receptors to directly interact with actin.

### Direct actin binding can overcome Ub-mediated sorting at the endosome

**Figure 7.**
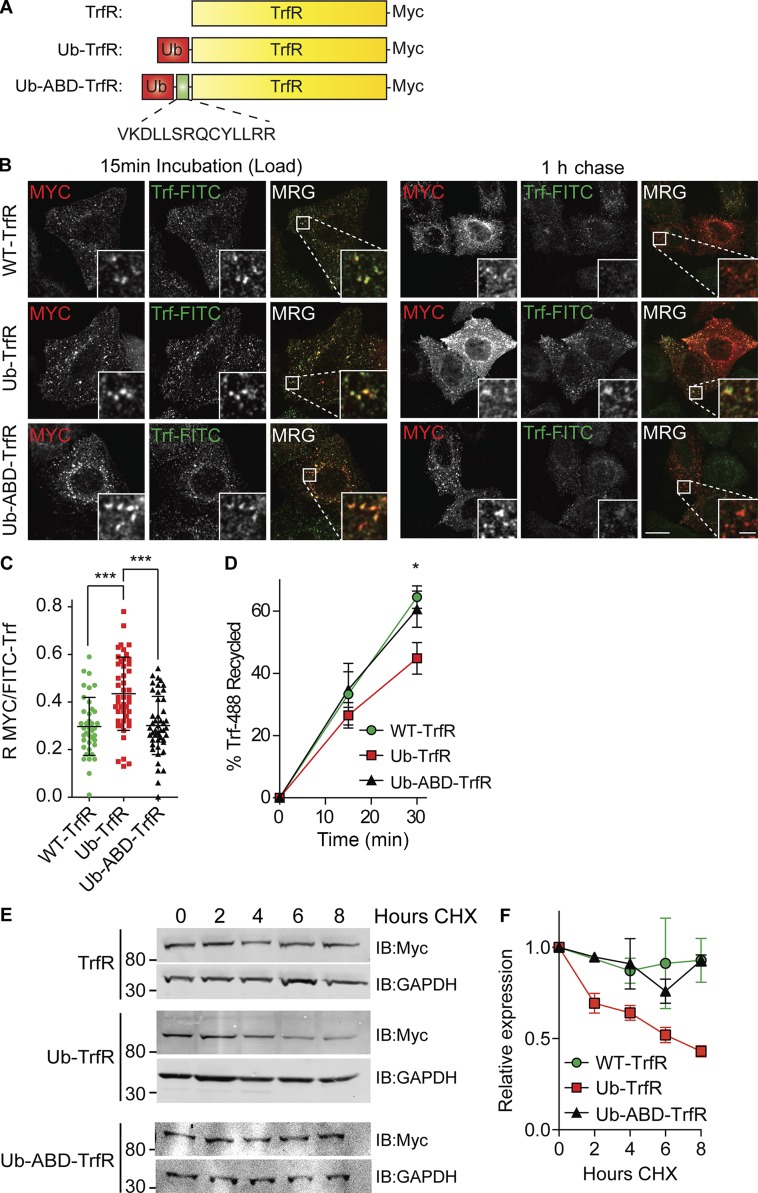
**Actin binding can overcome Ub sorting at the endosome. (A)** Schematic of TrfR chimeras. **(B)** HeLa cells were transfected with indicated constructs and then incubated in 50 µg/ml FITC-Trf for 15 min. The cells were washed in PBS and incubated in fresh medium with unlabeled Trf to chase for 1 h before fixation and staining. MRG, merge. **(C)** Pearson’s R values between the receptor (myc) and FITC-TrfR were calculated using Slidebook software (10 images approximately >150 cells total). *n* = 3. Error bars indicate SD. Bars: (main images) 10 µm; (insets) 2 µm. **(D)** HeLa S3 cells were transfected with the TrfR chimeras and treated as before except the recycling was stopped at 0, 15, and 30 min, and the retained FITC-Trf was measured by flow cytometry (*n* = 5). **(E and F)** HeLa cells were transfected with indicated fusion constructs for 24 h before treatment with CHX to block protein synthesis for the indicated time course. *n* = 3. Error bars indicate SEM. All experiments were performed in the presence of 50 µg/ml leupeptin and 100 µg/ml CHX except E and F, where leupeptin was omitted. *, P < 0.05; ***, P < 0.001. Statistical analysis, one-way ANOVA for all comparisons, with Dunnett’s post hoc test. Images taken from a single slice.

Our findings showing that HRS governs receptor recycling contrasts with a body of previous work linking it to the active sorting of ubiquitylated receptors into the lumen of MVBs (Raiborg and Stenmark, 2009). Sorting toward degradation or recycling is a crucial decision in receptor transport. We wanted to examine whether there is competition between actin binding required for recycling and Ub-mediated sorting into MVBs. To achieve this, we used a previously described assay (Raiborg et al., 2002) where a noncleavable moiety of ubiquitin is fused to the intracellular tail of TrfR. Normally TrfR is efficiently recycled; however, the fusion of a single Ub moiety is sufficient to sort the receptor via HRS and receptor sorting toward degradation. We added the actin-binding region of the cytoplasmic tail of MT1-MMP to the TrfR adjacent to a noncleavable ubiquitin (Ub) moiety (Ub-ABD-TrfR), creating a direct competition for the sorting of the chimeric receptor between Ub and actin-mediated sorting (Fig. 7 A).

HeLa cells were transfected with the WT TrfR, Ub-TrfR conjugate, or Ub-ABD-TrfR (Fig. S5 B). Transfected cells were incubated for 15 min with FITC-coupled Trf to achieve equilibrium loading (Fig. 7 B). Fresh media with unlabeled Trf were added to the cells, and the receptor ligand complexes were allowed to recycle for 1 h. The Ub-TrfR construct was retained in endosomes, and the construct showed a higher degree of colocalization with FITC-Trf after 1 h chase compared with the WT or Ub-ABD-TrfR (Fig. 7 C). This mirrored results previously reported with the Ub-TrfR construct, whereas the WT and Ub-ABD-TrfR recycled back to the plasma membrane and released the FITC-Trf as observed by a significantly reduced association with FITC-TrfR (Fig. 7, B and C; TrfR R, 0.29; Ub-TrfR R, 0.435; Ub-ABD-TrfR R, 0.301).

To characterize the dynamics of this sorting event, we used a flow cytometry assay. HeLa cells were transfected and loaded with FITC-Trf for 15 min before removal and fixation at the indicated time points and analysis of fluorescence intensity by flow cytometry. After 30 min, there was a 30.8% (±6.3%) increase in retention of the Ub-TrfR compared with WT receptor, whereas the ABD containing Ub-ABD-TrfR construct had identical recycling dynamics as the WT receptor (Fig. 7 D). We next tested whether the chimeric receptors had been redirected into the degradative pathway by the addition of the noncleavable Ub. To do this, we performed cycloheximide (CHX) chases blocking protein synthesis. The Ub-TrfR had a significantly increased rate of degradation compared with the WT receptor over an 8-h time course, indicating that the receptor is being sorted toward lysosomal degradation. In contrast, the addition of the ABD was able to rescue the effect of coupling Ub to the TrfR with the chimeric receptor having identical degradation rates as the WT receptor (Fig. 7, E and F). Our data demonstrate that actin binding can overcome monoubiquitin-mediated endosomal sorting.

### WASH–HRS axis is required for cell invasion

**Figure 8.**
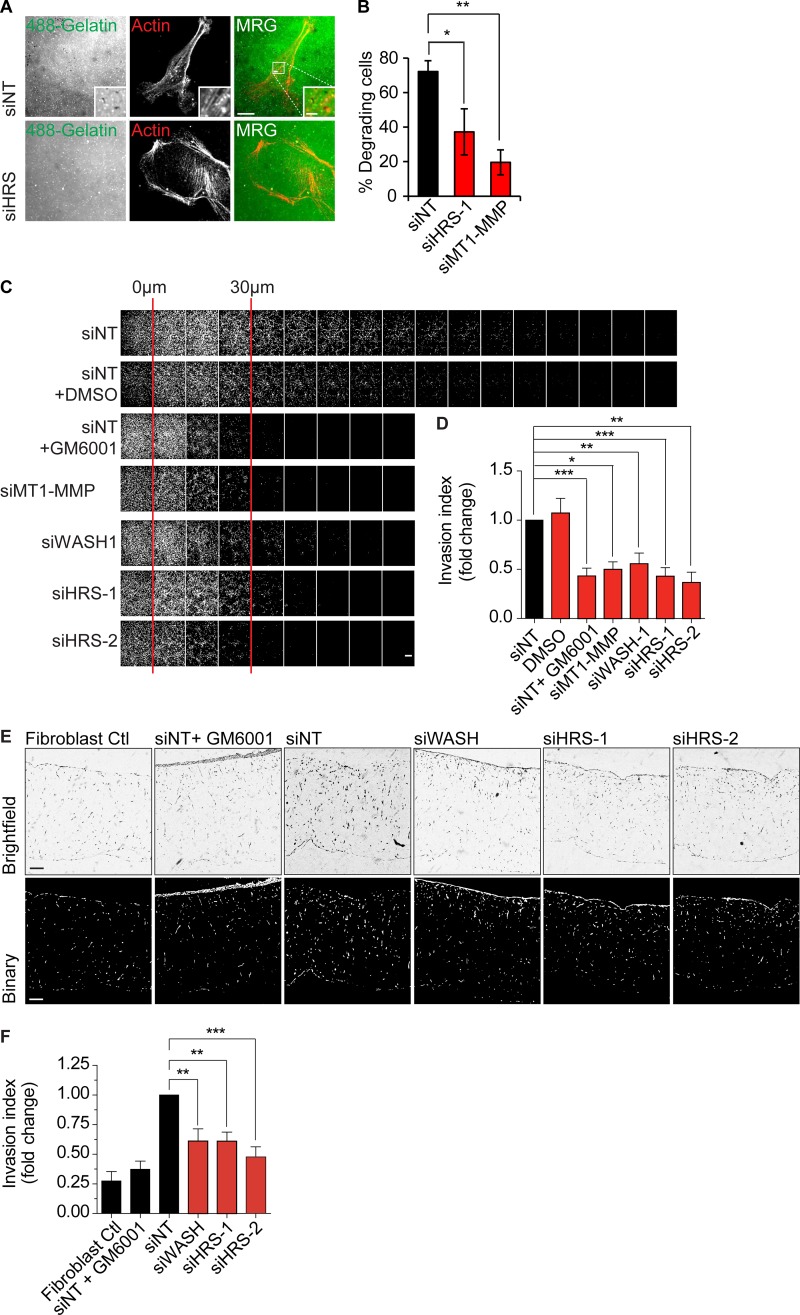
**HRS and WASH axis are required for matrix degradation and breast cancer cell invasion. (A)** MDA–MB-231 cells were treated with siRNA for 120 h before being seeded onto 488-gelatin–coated coverslips for 16 h. MRG, merge. **(B)** Percentage of degrading cells (*n* = 3 individual experiments; approximately >60 cells total). Images taken from a single slice. **(C)** MDA–MB-231 cells were treated with siRNA for 96 h before 8 × 10^4^ cells seeded for an inverted invasion assay on a gel composed of Matrigel/fibronectin followed by culture for 5 d. Images were taken every 10 µm and analyzed using ImageJ. **(D)** Relative invasion index over 30 µm (*n* ≥ 3). 20 ng/ml EGF was used as a chemoattractant. GM6001 (5 µM), DMSO (1:1,000), and gels without cells were used as invasion, vehicle, and fibroblast background controls, respectively. **(E)** Organotypic raft culture. MDA–MB-231 cells were treated with siRNA for 96 h before 7 × 10^5^ cells seeded on fibroblast remodeled collagen gels and allowed to adhere for 3 d. Gels were then cultured at the liquid–air interface for a further 7 d. Gels were fixed, embedded, hematoxylin and eosin–stained, and sectioned to determine invasion index. Bars: (main images) 10 µm; (insets) 2 µm. **(F)** Quantification of MDA–MB-231 cell invasion relative to siNT control (*n* = 4 independent experiments). Error bars indicate SEM. *, P < 0.05; **, P < 0.01; ***, P < 0.001. Statistical analysis, one-way ANOVA for all comparisons with Dunnett’s post hoc test.

MT1-MMP is a pivotal MMP required for degradation of matrix proteins to enable cancer cell invasion into nonpermissive extracellular matrix (Hotary et al., 2003, 2006; Sabeh et al., 2004). We sought to test whether HRS-dependent recycling has a functional role in cancer cell properties that could be ascribed to MT1-MMP dynamics. We analyzed whether the HRS–WASH axis was required for MT1-MMP–dependent triple-negative breast cancer cell matrix degradation, migration, and invasion. To assess invadopodia-based degradation ability, MDA–MB-231 cells were depleted of HRS using siRNA and seeded onto coverslips coated with labeled gelatin overnight. There was a significant decrease in the ability of HRS-depleted cells to degrade gelatin over 16 h (Fig. 8, A and B). We observed no change in the ability of MDA–MB-231 cells to migrate over plastic, indicating that the cells maintained the essential migration machinery (Fig. S5, C–F). An inverted invasion assay, into a gel composed of Matrigel and fibronectin, showed a significant decrease in invasion capacity of HRS- and WASH-depleted cells, which was indistinguishable from cells treated with the metalloproteinase inhibitor GM6001 or MT1-MMP knockdown (Fig. 8, C and D). Cross-linked collagen I provides a more realistic substrate barrier for cancer cell invasion, with the pore size inhibiting any migration unless there is accompanying matrix degradation (Wolf and Friedl, 2009; Wolf et al., 2009). MDA–MB-231 cells were depleted of HRS or WASH and seeded onto fibroblast-remodeled organotypic collagen gels for 2–3 d before culture at the liquid–air interphase for 7 d. The silencing or HRS and WASH significantly reduced invasion by 40–50% (Fig. 8, E and F). These experiments support the model that the HRS–WASH axis is important for breast cancer cell invasion.

## Discussion

HRS is constitutively associated with STAM to form the core of the ESCRT-0 complex. A body of data exists linking this complex to the capture of ubiquitylated proteins and to recruitment of the ESCRT-I complex. It is a key element of the ESCRT machinery devoted to directing proteins into MVBs for transport to lysosomes. Other aspects of HRS function have also been reported. It has been described to dictate the retrograde trafficking of the β-adreno and tropomyosin receptor kinase B receptors through an unknown mechanism that depends on the expression of the VHS and FYVE domains of HRS (Hanyaloglu and von Zastrow, 2007; Huang et al., 2009). In this study, we uncover a key role for HRS in the endosomal association and activity of the actin polymerization factor WASH. We show that this axis governs the recycling to the plasma membrane of proteins that contain defined actin-binding motifs (EGFR and MT1-MMP).

WASH complex localization had previously been defined to span Rab4-, EEA1-, and Rab7-positive endosomes, which are considered to be receptor recycling–competent (Zech et al., 2011; Dozynkiewicz et al., 2012; Macpherson et al., 2014). Previous studies have shown a role for the retromer component VPS35 in the recruitment of WASH to endosomes (Harbour et al., 2010, 2012; Jia et al., 2012). Direct interactions of VPS35 and the retromer-associated sorting nexins 1 and 3 with HRS have been reported that could potentially provide a link to the WASH complex (Pons et al., 2008; Popoff et al., 2009). Despite a decrease in the total pool of VPS35 in HRS depleted HeLa cells, we saw no changes in the endosomal pool of VPS35 in HeLa and MDA–MB-231 cells. This is in agreement with studies showing at least partial retromer-independent endosomal WASH recruitment in *Dictyostelium discoideum* (Park et al., 2013) and mouse VPS35 knockout cells (McNally et al., 2017). A recent study has identified a retromer-analogous complex called retriever that is required for retrograde trafficking of WASH-dependent cargo, for example α5β1 integrin, through a WASH-FAM21-CCC complex–retriever cascade (Bartuzi et al., 2016; McNally et al., 2017). We did not observe a reduction in endosomal levels of the CCC complex member COMMD1 after HRS depletion, but it will be interesting to see whether HRS and the interplay with the degradation pathway will have an impact on endosomal retriever dynamics and activity.

Rather than being a retromer-only dependent recruitment process for WASH on the endosome, we found that a minimal construct of HRS encompassing a FYVE domain and adjacent VHS domain (VHS-FYVE) is sufficient for WASH recruitment to endosomes. The FYVE domain of HRS is necessary for its recruitment to endosomes through binding to the inositol lipid PtdIns3*P*, whose levels we find to be unchanged after HRS depletion (Urbé et al., 2000; Raiborg et al., 2001). The VHS domain has a less-clear function. It forms a “superhelix” of eight α helices that can behave as a multipurpose docking site capable of binding to membranes and proteins (Mao et al., 2000). The VHS domains in GGA proteins have been shown to directly interact with cargo (Puertollano et al., 2001; Misra et al., 2002), whereas the VHS-FYVE domain of HRS can directly bind to Ub chains (Ren and Hurley, 2010). Although we have no evidence for direct HRS–WASH complex binding, we speculate that this minimal component can either recruit an adapter protein or otherwise configure endosomal domain architecture to enable binding.

**Figure 9.**
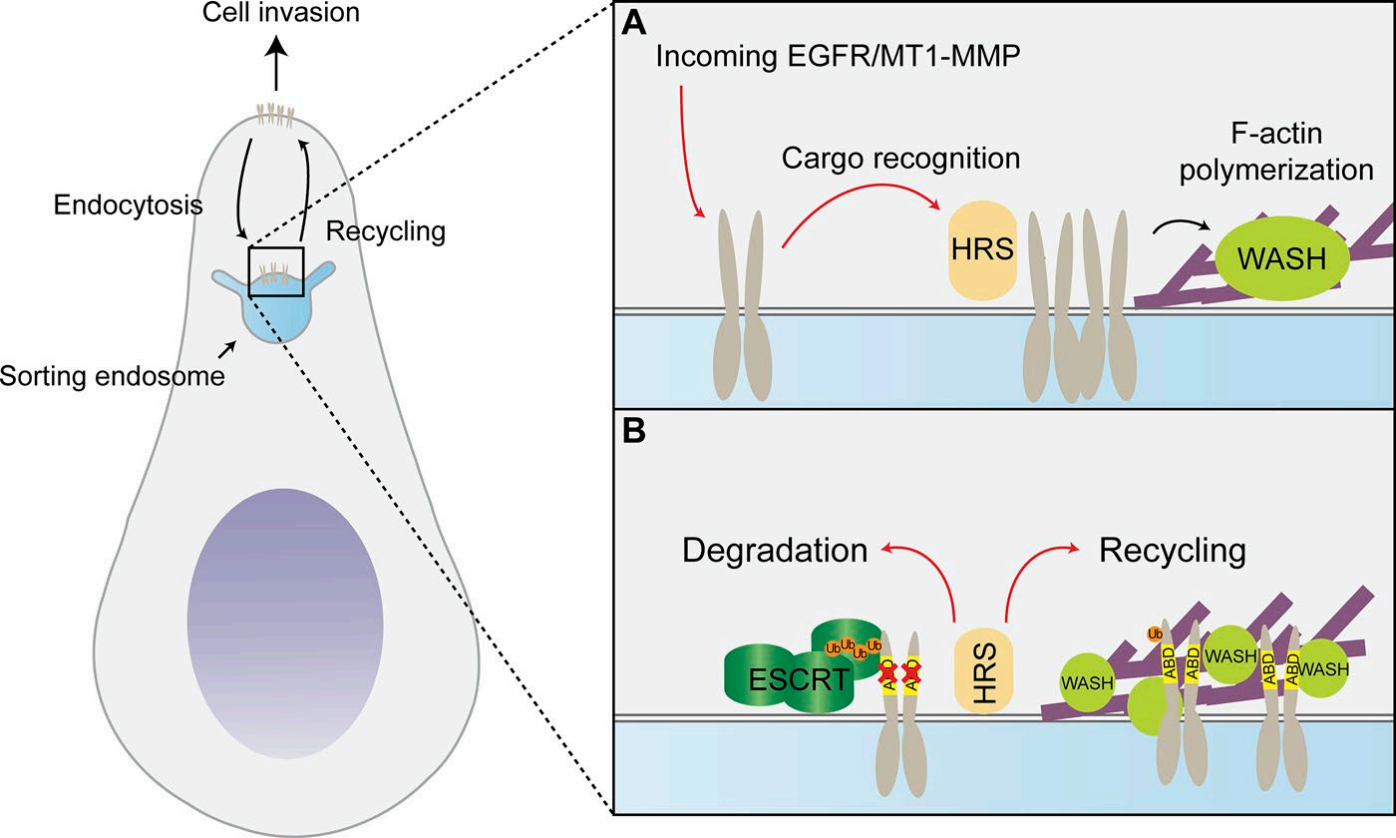
**Model. (A)** HRS is required for WASH recruitment and actin polymerization on the endosome. **(B)** Actin corrals receptors into an actin meshwork on the endosome that sequesters it into a recycling domain and enables efficient recycling. Receptor–actin interactions need to be disrupted before ESCRT-driven degradation can occur.

We confirmed functional consequences of HRS governance over WASH recruitment by showing a requirement for HRS in the constitutive recycling of the WASH-dependent cargos EGFR, ci-M6PR, and MT1-MMP. The HRS–WASH axis facilitated recycling of EGFR and MT1-MMP through a mechanism that required direct actin binding of the receptors at the endosome. This introduces a new principle for sequence dependent sorting at the endosome that may extend to other recycling components known to be able to indirectly interact with actin such as integrins (Calderwood et al., 2000; Jiang et al., 2003). Previously, actin on endosomes has been shown to provide a mode for stabilizing tubules, allowing more time for receptors to be concentrated (Puthenveedu et al., 2010). Although the WASH complex binds to the fission machinery through dynamin for the pinching off of new vesicles from the sorting endosome (Derivery et al., 2009), we propose that endosomal F-actin function could include sequestering receptors into discrete recycling subdomains on the limiting membrane, concentrating receptors and enabling their efficient recycling (Fig. 9; Puthenveedu et al., 2010; Zech et al., 2012). This hypothesis is supported by our chimera experiments using a TrfR scaffold, where the inclusion of the ABD from MT1-MMP could overcome Ub-mediated sorting by the ESCRT complex (Raiborg et al., 2002). These three levels of actin involvement on the endosome provide a coherent set of steps that require actin involvement in receptor trafficking from cargo sorting to fission of the vesicle on a recycling-ready endosome.

How can we reconcile the fact that EGFR ubiquitylation provides a signal for degradation yet incorporates an actin-binding motif that can deflect from this pathway? Our chimera experiments are conducted with a single Ub moiety providing the lysosomal sorting signal. In reality, acutely activated EGF receptors are decorated with multiple Ub molecules through monoubiquitylation and Ub chains (Huang et al., 2006). Thus, we propose that under steady-state conditions, where EGFR is internalized but not tagged for degradation, the actin-binding motif may ensure efficient recycling and/or deposition at a specific area of the plasma membrane. Under conditions of strong acute stimulation that lead to receptor degradation, the cooperativity of multiple Ub interactions within the ESCRT-0 and -I complexes may ensure that the ESCRT–MVB pathway is engaged to a larger extent.

However, a central conundrum remains, in that both opposing pathways require receptor interactions with HRS. It is possible that the VHS domain of HRS, like its equivalent in GGA proteins, can bind to both ubiquitylated and nonubiquitylated proteins because binding has only ever been tested with ubiquitylated proteins (Ren and Hurley, 2010). Another possibility invokes the enigmatic function of the phosphorylation of HRS (Row et al., 2005). HRS was originally identified as a prominent substrate of receptor tyrosine kinases (Komada and Kitamura, 1995). It could be that after engagement of activated receptors, the consequent HRS phosphorylation disables the recycling function of HRS by its removal from the endomembrane (Urbé et al., 2000) to ensure effective degradation.

Cancer cell invasion is dependent on recycling of proteins required for matrix degradation and interactions (Caswell and Norman, 2008; Castro-Castro et al., 2016). We found that HRS and actin binding of MT1-MMP are necessary for recycling of MT1-MMP. We have demonstrated the functional importance of this axis by the blockade in breast cancer cell invasion with loss of HRS–WASH. Depletion of HRS function did not result in changes in random migration on 2D substrates, and thus the basic migration machinery of the cell remains intact. In contrast, invasion into 3D collagen organotypic raft culture and the associated ability of cells to degrade matrix was abrogated. The invasive migration of triple-negative MDA–MB-231 breast cancer cells through dense matrix has been shown to depend on the function and localization of MT1-MMP in invasive pseudopods (Monteiro et al., 2013; Castro-Castro et al., 2016). We propose that failure of MT1-MMP recycling when HRS–WASH are lost explains this loss of invasive capability with intact migration capacity, demonstrating the functional importance of this axis in invasive migration.

## Materials and methods

### Materials

The following antibodies were used in this study (specific applications indicated where two antibodies against the same target are noted): anti-EEA1 (610457; BD), anti-EGFR (555996; BD; recycling assays), anti-EGFR (4267; immunofluorescence [IF]; Cell Signaling Technology), anti-EGFR (ab52894; rabbit IF; Abcam), anti-p34 (07–227; EMD Millipore), anti–cathepsin D (219361; EMD Millipore), CCDC53 (HPA038338; Atlas), strumpellin (ab101222; EMD Millipore), MT1-MMP antibody (MAB3328; EMD Millipore), anti-VPS35 (Ab10099; Abcam), anti-WASH (HPA002689; Atlas), anti-HRS (EBO7211; Western blot; Everest), anti-HRS (ALX-804-382-C050; IF), anti-STAM (homemade [Row et al., 2005]), anti-CCDC53 (ABT69; EMD Millipore), anti-Myc (05-274; Merck), anti-ciM6PR (ab2733; Abcam), anti-TGN46 (T7576; Sigma-Aldrich), COMMD1 (11938–1-AP; ProteinTech), rabbit anti-TrfR (ab84036; Abcam), mouse anti-TrfR (ab38171; Abcam), anti–α-tubulin (T5168; Sigma-Aldrich), and anti-GAPDH (AB2302; EMD Millipore). Donkey IR700 and IR800-coupled anti–mouse and anti–rabbit secondary antibodies were purchased from LI-COR Biosciences, and Alexa Fluor 488–, Alexa Fluor 594–, and Alexa Fluor 647–coupled donkey anti–mouse, anti–goat, and anti–rabbit antibodies were obtained from Molecular Probes. Acti-stain 670 phalloidin (Cytoskeleton, Inc.) and FITC-Trf (T2871; Molecular Probes) were also used.

The following siRNAs were used in this study: NT, Allstar negative nontargeting control 2 (Qiagen); WASH-1, 5′-GCCACAGGAUCCAGAGCAA-3′ (dTdT); HRS-1, 5′-CGUCUUUCCAGAAUUCAAA-3′ (dTdT; S17480; Ambion); HRS-2, 5′-UGGAAUCUGUGGUAAAGAA-3′ (dTdT; s17481; Ambion); and MT1-MMP target sequence: 5′-GACAGCGGTCTAGGAATTCAA-3′.

All other reagents were acquired from Sigma-Aldrich unless otherwise stated.

### Cell culture and transfection

HeLa, HeLa S3 (Flp-In), and MDA–MB-231 cells were cultured at 37°C in 5% CO_2_ in DMEM supplemented with 10% FBS, 1% nonessential amino acids, and 1% penicillin/streptomycin sulfate. Flp-In HeLa S3 stably transfected cells were supplemented with 150 µg/ml hygromycin B. For siRNA transfections, all cells were treated with siRNA to a final concentration of 50 nM twice over 120 h at 0- and 48-h time points. HeLa and HeLa S3 (Flp-In) cells were treated using RNAiMAX transfection reagent (Invitrogen), and MDA–MB-231 cells were treated using Lullaby transfection reagent (OZ Bioscience). For plasmid expression, all cells were transfected using Lipofectamine 2000 in a ratio of 1 µg DNA:3 µl Lipofectamine 2000 for one well of a six-well plate.

### Generating stable cell lines

A Flp-In HeLa S3 stable host cell line was generated according to the Flp-In system manufacturer’s (Invitrogen) instructions and verified by Northern blotting (unpublished data). GFP and GFP-HRS were amplified from pEGFP-C1-HRS (mouse) with the following primers: forward, both with 5′-CACCATGGTGAGCAAGGGCG-3′; and reverse, GFP with 5′-TTAGGATCTGAGTCCGGACTTGTACAGC-3′ and GFP-HRS with 5′-GGCCCGCGGTACCGTCGA-3′. PCR products were then cloned into pEF5/FRT/V5 TOPO using a pEF5/FRT/V5 Directional TOPO cloning kit (Thermo Fisher Scientific) resulting in pEF5/FRT/V5-GFP and pEF5/FRT/V5-GFP-HRS plasmids. To generate stable cell lines, HeLa S3 Flp-In host cells were transfected with pEF5/FRT/V5-GFP and pEF5/FRT/V5-GFP-HRS together with pOG44 plasmid, which expresses the Flp recombinase at a ratio of 1:9. After hygromycin B selection (200 µg/ml), single colonies were picked and grown up separately as individual clones.

### Microscopy

Cells for regular IF were fixed in 4% PFA/PBS, quenched in 50 mM NH_4_Cl, and permeabilized in 0.2% Triton X-100/PBS. Blocking and antibody labeling was performed in 5% donkey serum. For guanidinium hydrochloride denaturing–based staining, the same protocol was followed with the addition of 10-min incubation in 6 M guanidinium hydrochloride followed by three washes in PBS after permeabilization. All images were recorded with a Marianas spinning disk confocal microscope (3i) using a 63× 1.4 NA or 10× 0.45 NA Zeiss Plan Apochromat lens and either an Evolve electron-multiplying charge-coupled device (Photometrics) or FLASH4 sCMOS (Hamamatsu) camera or Zeiss LSM800 with Airyscan module, using a 63× 1.4 NA Zeiss Plan Apochromat. Single-cell migration assays were performed on a Nikon Ti-E using a 20× CFI Super Plan Fluor extra-long working distance ADM 0.45 NA and a CoolSnap HQ camera (Photometrics).

paCherry and paGFP experiments were performed by exposure of Rab4- or EEA1-positive endosomes to a brief pulse of 405-nm laser light. A time sequence was acquired. The intensity of fluorescence in the endosome was quantified and normalized to background photobleaching and peak endosomal fluorescence. Rate of decay of fluorescence (k = s^−1^) was extracted from curves fitted using the one-phase dissociation equation in Prism (GraphPad Software). For FRAP, experiments a one-phase association curve was fitted to the data, and the rate constant k = s^−1^ was extracted. Slidebook software (3i) was used to quantify colocalization, FRAP, and paGFP photoactivation experiments. Zeiss Zen software was used to process Airyscan images, and the ImageJ (National Institutes of Health) FRAPprofiler plugin was used to quantify paCherry photoactivation experiments. For WASH/EEA1 in Flp-In cells, EGFR/EEA1, and VPS35/EEA1, a mask was generated around EEA1- or TGN46-positive structures, the background was subtracted, and the colocalization of EEA1 and WASH was calculated as R. For ciM6PR experiments, colocalization was calculated from the whole cell using either Slidebook software of ImageJ. Sum intensity measurements on endosomes were quantified by making a mask around the endosome (EEA1 or VPS35), then calculating the sum intensity using the Slidebook software in the endosome divided by the volume of the endosome. Calculation of colocalization with EEA1 (GFP-VHS-FYVE rescue experiments and HRS rescue experiments) was performed by generating a mask around EEA1 calculating the R value in the Slidebook. Thresholds were set the same for all images. For TrfR recycling assay, a mask was generated around the Myc-TrfR signal, and the R value against Trf-488 was measured. For actin quantification on endosomes, a mask was generated around VPS35-positive structures, and the intensity of actin was measured in the mask. Single-cell tracking was analyzed using the manual tracking and chemotaxis plugin in ImageJ. Identical exposure settings where used in all experiments where comparisons were made between conditions.

### PLA

HeLa S3 cells were seeded on coverslips 24 h before assay. Cells were fixed with 4% PFA and permeabilized with 0.2% Triton X-100/PBS. PLAs was performed using the Duolink II reagent kit (Olink) according to the manufacturer’s specifications using blocking buffers and antibody diluent outlined above (see Microscopy), HRS and WASH antibodies were used at a dilution factor of 1:500. Either HRS or WASH antibodies alone were used as technical controls. Coverslips were imaged using the Marianas spinning disk confocal microscope (3i), 40× objective lens, and FLASH4 sCMOS camera. PLA signals were identified and counted using ImageJ and expressed as the number of signals/cells in the optical field.

### TrfR chimera assays

the TrfR chimera recycling assay was adapted from Raiborg et al. (2002). TrfR chimera constructs have been previously described (Raiborg et al., 2002). In brief, mouse Ub was fused to human TrfR construct with a spacer Arg-Ser-Gln-Gln and the omission of the carboxy tail glycine residues of Ub to prevent the removal of Ub by deubiquitinases. The ABD from MT1-MMP was inserted between TrfR tail and ubiquitin moiety by annealing the following primers: 5′-TCTCAGAAGATCACAACAGGTCAAGGACCTGCTGTCCCGTCAGTGCTACCTCCTGCGAAGGATGATGGAT-3′; and 5′-ATCCATCATCCTTCGCAGGAGGTAGCACTGACGGGACAGCAGGTCCTTGACCTGTTGTGATCTTCTGAG-3′.

The whole construct was generated by PCR reaction using the following primers and pcDNA3-6-HIS-Ub, pcDNA3-hTrFR vectors provided by C. Raiborg (Center for Cancer Biomedicine Norwegian Radium Hospital, Oslo, Norway) Ub_F, 5′-CTGGATCCATGCAGATCTTCGTGAAGACT-3′; TrfR, 5′-CGTTTGGGACATTGACAATGAGTTTTAAACTAGTGAATTCAT-3′; Ub-MTMMP1(tail)fusion, 5′-CCTGGTGCTCCGTCTCAGAAGATCACAACAGGTCAAG-3′; 5′-CTTGACCTGTTGTGATCTTCTGAGACGGAGCACCAGG-3′; MTMMP1(Tail)-TrfR fusion, 5′-GCTACCTCCTGCGAAGGATGATGGATCAAGCTAGATCAGCA-3′; 5′-TGCTGATCTAGCTTGATCCATCATCCTTCGCAGGAGGTAGC-3′.

The constructs were then subcloned into pDM734 vector to add a myc tag.

HeLa and HeLaS3 cells were transfected with TrfR constructs for 24 h before incubation with 50 µg/ml FITC-Trf for 15 min. The cells were washed in warm PBS and then allowed to recycle the TrfR construct in fresh full DMEM supplemented with 50 µg/ml leupeptin and 100 µg/ml CHX. Cells were fixed in 4% PFA before processing for IF or flow cytometry. For analysis by flow cytometry after TrfR construct transfection as described above, cells were serum starved for 1–2 h before harvesting by lifting cells with 5 mM EDTA at 37°C. Cells were washed 3× in PBS and resuspended in serum-free medium for incubation on ice for 30 min. Cells were then incubated with 37°C full DMEM supplemented with 50 µg/ml FITC-Trf for 30 min at 37°C before being returned to ice. Cells were washed 3× with ice-cold PBS before incubation in 37°C full DMEM supplemented with 50 µg/ml leupeptin and 100 µg/ml CHX for the indicated recycling time points. After incubation, cells were returned to ice and washed 3× with ice-cold PBS before fixation in 4% PFA for 30 min. Cells were permeabilized with 0.1% Triton X-100 for 15 min, washed, and blocked with 1% BSA for 15 min. To determine the mean fluorescence intensity of FITC-Trf in cells expressing TrfR constructs, cells were labeled with mouse anti-Myc (EMD Millipore) primary antibody followed by Alexa Fluor 594 anti–mouse secondary antibody (Invitrogen). FITC-Trf mean fluorescence intensity of Alexa Fluor 594–positive cells was determined using an Attune NxT Flow cytometer (Thermo Fisher Scientific).

### ci-M6PR secretion assay

The assay was performed as described in MacDonald et al. (2014). In brief, siRNA treated HeLa cells were washed in PBS and incubated overnight in OptiMEM (GIBCO BRL; Thermo Fisher Scientific). The conditioned media was collected. Protein content was TCA precipitated, and the resulting pellet was resuspended in SDS running buffer. The underlying cells were lysed in NP-40 buffer.

### EGFR trafficking assays

The EGFR trafficking assay was performed as previously described in Roberts et al. (2001). In brief, siRNA-treated MDA–MB-231 cells were seeded into 10-cm dishes and grown to subconfluency on the day of the experiment. Cells were serum-starved 1 h before recycling in serum-free DMEM. Cells were biotinylated on ice in 10 mg sulfo-*N*-hydroxysuccinimide–SS-biotin per 75 ml PBS (21331; Thermo Fisher Scientific). Cells were then placed in serum-free DMEM 37°C for 30 min to internalize receptor–biotin complexes to equilibrium. Remaining cell surface biotin was stripped in 92-mM 2-mercaptoethanesulfonate sodium. Cells were placed again in serum-free DMEM for defined recycling periods at 37°C and subsequently stripped again and quenched with iodoacetamide. Cells were scraped and syringed in lysis buffer (200 mM NaCl, 75 mM Tris, 15 mM NaF, 7.5 mM EDTA, and EGTA, 1.5% Triton X-100, 0.075% Igepal CA-630, and Halt protease and phosphatase inhibitors [Thermo Fisher Scientific]). The levels of biotinylated receptor were measured using a sandwich ELISA with anti-EGFR antibody, streptavidin–horseradish peroxidase, and 0.56 mg/ml orthophenylenediamine. The percentage of recycled receptor was quantified as a percentage of the internal pool.

### EGFR cell surface expression

In brief, after siRNA transfection (120 h), cells were harvested using 5 mM EDTA/PBS at 37°C. Cells were washed 3× in PBS and fixed in 4% PFA for 30 min, washed, and blocked using 2% BSA/PBS supplemented with 0.1% sodium azide for 15 min. Cells were labeled with rabbit anti-EGFR primary antibody followed by Alexa Fluor 488 anti–rabbit secondary antibody. Mean fluorescence intensity of Alexa Fluor 488–positive cells was determined using an Attune NxT Flow cytometer (Thermo Fisher Scientific).

### Cell lysis for Western blotting

Cells were washed 2× in ice-cold PBS and lysed as indicated in NP-40 buffer (0.5% NP-40, 25 mM Tris, pH 7.5, 100 mM NaCl) or radioimmunoprecipitation assay (RIPA) buffer (10 mM Tris-HCl, 150 mM NaCl, 1% Triton X-100 [wt/vol], 0.1% SDS [wt/vol], and 1% sodium deoxycholate [wt/vol]) supplemented with Halt protease and phosphatase inhibitors (Thermo Fisher Scientific) by rocking at 4°C for 10 min.

### Gelatin degradation assay

siRNA-treated MDA–MB-231 cells were seeded for 16 h onto 488-gelatin–covered coverslips. The cells were fixed in 4% PFA/PBS and processed for IF. Degradation was quantified by counting cells that had actin-positive degradation spots below the cell.

### Invasion assays

Inverted invasion assays were performed as described previously (Hennigan et al., 1994). In brief, Matrigel (Corning) was diluted with PBS to 5 mg/ml, supplemented with fibronectin to a final concentration of 25 µg/ml, and polymerized in transwell inserts (Corning) at 37°C for 1 h. Inserts were inverted, and 8 × 10^4^ cells were seeded directly to the bottom of the filter. MDA–MB-231 cells were seeded and allowed to adhere for 3–6 h. Once adhered, the inserts were turned right side up. Serum-free medium was added to the wells of the transwell plate, and medium supplemented with 10% FBS and 20 ng/ml EGF was added on top of the Matrigel. After a 5-d incubation, gels were fixed in 4% PFA for 30 min, followed by permeabilization with 0.1% Triton X-100 in PBS for 30 min. Samples were then stained with DAPI (Sigma-Aldrich) for 1 h or overnight at 4°C. Cells failing to cross the filter were removed with tissue. Serial optical sections of the plug at 10-µm intervals using an inverted spinning disk confocal microscope (Marianas; 3i) fitted with a 10 × 0.45 NA air objective lens were taken. ImageJ was used to determine the integrated density of each optical section to determine the invasion index = (∑ integrated density of first 30 µM)/−(∑ integrated density of invasion) and expressed as fold change with respect to the NT control as performed in Zech et al. (2011).

Organotypic raft cultures were previously described (Timpson et al., 2011). In brief, at 4°C immortalized human mammary fibroblasts (8 × 10^4^/ml) were resuspended in Type 1 rat tail collagen (∼1 mg/ml) supplemented with 10% FBS and 1× DMEM, pH 7.2, and plated into 35-mm dishes (2.5 ml/dish). Collagen was allowed to polymerize for 15–30 min at 37°C before adding 1–2 ml of full DMEM supplemented with Hepes. Media were changed every other day until collagen contracted to ∼1.5 cm in diameter. Once contracted, gels were placed into a 24-well plate followed by 7 × 10^5^ cells in suspension. Cells were allowed to adhere for 3 d, after which gels were lifted onto stainless steel grids in 6-cm dishes. Media supplemented with 20 ng/ml EGF was added so the bottom of the gel is in contact with media but not submerged. For negative invasion controls, GM6001 (5 µM) was added to the media. After a 5-d incubation, gels were cut in half and fixed in 4% PFA overnight before embedding, sectioning, and hematoxylin and eosin staining. Invasion index was determined as previously described (invasion index = mean invasive depth × number of particles × area of particles; Jenei et al., 2011).

### Online supplemental material

Figs. S1 and S2 show additional evidence that HRS is required for the endosomal recruitment of WASH and investigate expression levels and localization of associated proteins. Fig. S3 shows evidence that the HRS VHS-FYVE domains are sufficient to recruit WASH to endomembranes. Fig. S4 shows that HRS is required for ci-M6PR receptor recycling. Fig. S5 shows that loss of HRS does not affect cell migration on a 2D substrate. Video 1 shows HeLa S3 Flp-In cells stably expressing GFP-mHRS transfected with mCherry-mWASH on a WASH-depleted background. Video 2 shows photoactivation of endosomal EGFR-paGFP in HRS-depleted cells. HeLa cells treated with the indicated siRNA for 120 h were transfected with EGFR-paGFP and mCherry-RAB4. paGFP was activated in mCherry-positive endosomes using a pulse of 405-nm laser light. Video 3 shows photoactivation of endosomal EGFR-paGFP. HeLa cells were transfected with EGFR-paGFP/EGFR-YLIP/AAAA-paGFP (actin-binding mutant) and mCherry-RAB4. paGFP was activated in RAB4-positive endosomes using a pulse of 405-nm laser light. Video 4 shows cellular distribution of WT and actin-binding mutant–overexpressed EGFR-GFP. HeLa cells transfected with either EGFR-GFP or EGFR-YLIP/AAAA-GFP and mRFP-EEA1. Video 5 shows HeLa cells transfected with indicated siRNA for 120 h that were transfected with EGFR-paGFP and mCherry-RAB4. Cells were pretreated with 100 mM primaquine to inhibit recycling before paGFP was activated in mCherry-positive endosomes using a pulse of 405-nm laser light. Video 6 shows photoactivation of endosomal paCherry–MT1-MMP constructs. MDA–MB-231 cells were transfected with paCherry–MT1-MMP/paCherry–MT1-MMP–LLY/AAA (actin-binding mutant) and GFP-EEA1. paCherry was activated in EEA1-positive endosomes using a pulse of 405-nm laser light.

## Supplementary Material

Supplemental Materials (PDF)

Video 1

Video 2

Video 3

Video 4

Video 5

Video 6
