# Supplementary material for: HRS–WASH axis governs actin-mediated endosomal recycling and cell invasion
Source: J Cell Biol. 2018 Jul 2;217(7):2549–64. doi: 10.1083/jcb.201710051 (PMC6028553; doi:10.1083/jcb.201710051)
Supplement: Supplemental Materials (PDF) [file JCB_201710051_sm.pdf]

Supplemental material

MacDonald et al., https://doi.org/10.1083/jcb.201710051

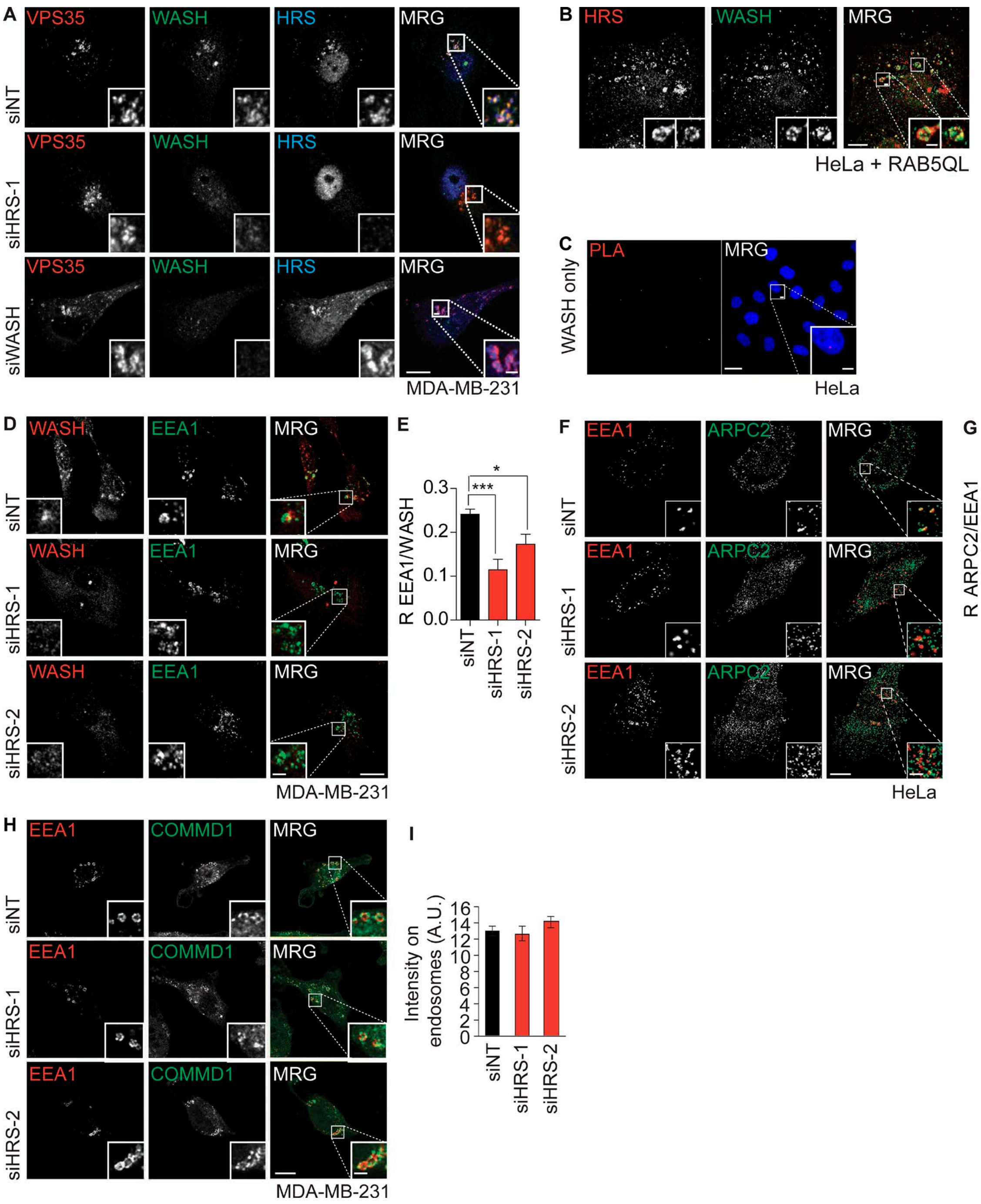

Figure S1. **HRS is required for the endosomal recruitment of WASH. (A)** MDA–MB-231 cells were treated with the indicated siRNA for 120 h before fixation in 4% PFA/PBS and labeling with antibodies. **(B)** HeLa cells were transfected with Flag-Rab5QL for 24 h before fixation in 4% PFA and labeling with the indicated antibodies. **(C)** PLA identifying protein–protein interactions between 10–40 nm. Technical control, WASH-only antibody. Maximum-projection image. **(D)** MDA–MB-231 cells were treated with the indicated siRNA for 120 h before fixation in 4% PFA/PBS and labeling with antibodies. **(E)** Pearson's R correlation value for WASH and EEA1 (~10 images per condition; >150 cells total). **(F)** HeLa cells were treated with the indicated siRNA for 120 h before fixation in 4% PFA/PBS and labeling with antibodies. **(G)** Pearson's R value for EEA1 and ARPC2 (30 cells total). **(H)** MDA–MB-231 cells were treated with the indicated siRNA for 120 h before fixation in 4% PFA/PBS and labeling with antibodies. MRG, merge. **(I)** Sum intensity value for COMMD1 on EEA1 endosomes (A.U.; ~10 images per condition; 30 cells total). *n* = 3; error bars indicate SEM. *, $P < 0.05$; ***, $P < 0.001$. Statistical analysis, one-way ANOVA for all comparisons with Dunnett's post hoc test. Bars: (main images) 10 µm; (insets) 2 µm. Images show a single slice, except C.

JCB

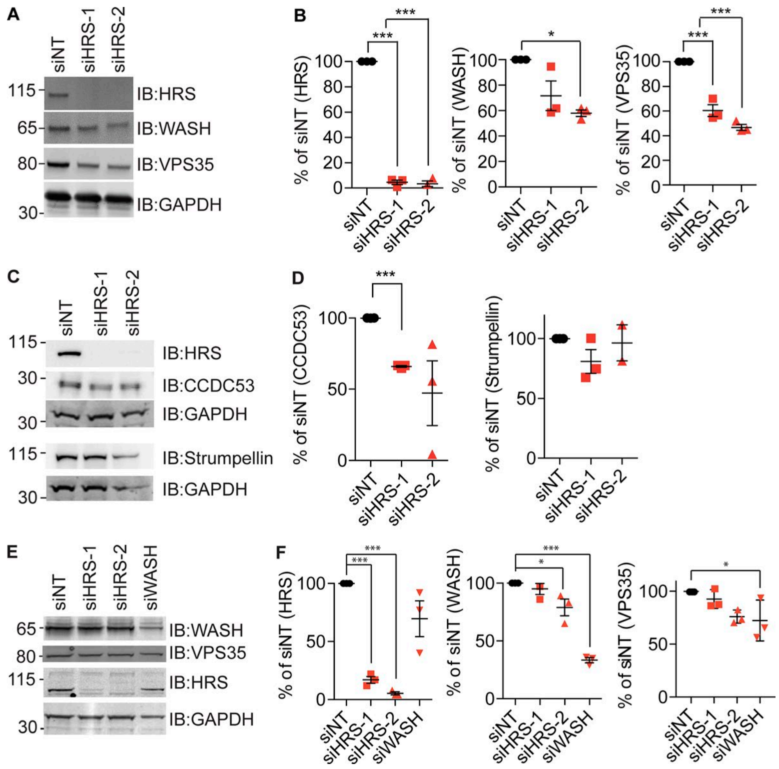

Figure S2. **HRS is required for the endosomal recruitment of WASH. (A–F)** HeLa cells (A–D) and MDA-MB-231 cells (E and F) were treated with siRNA for 120 h before lysis in RIPA buffer. $n = 3$. *, $P < 0.05$; ***, $P < 0.001$. One-way ANOVA with Dunnett's post hoc test. Western blot band intensities were quantified from Odyssey scans. IB, immunoblot. Molecular masses are given in kilodaltons.

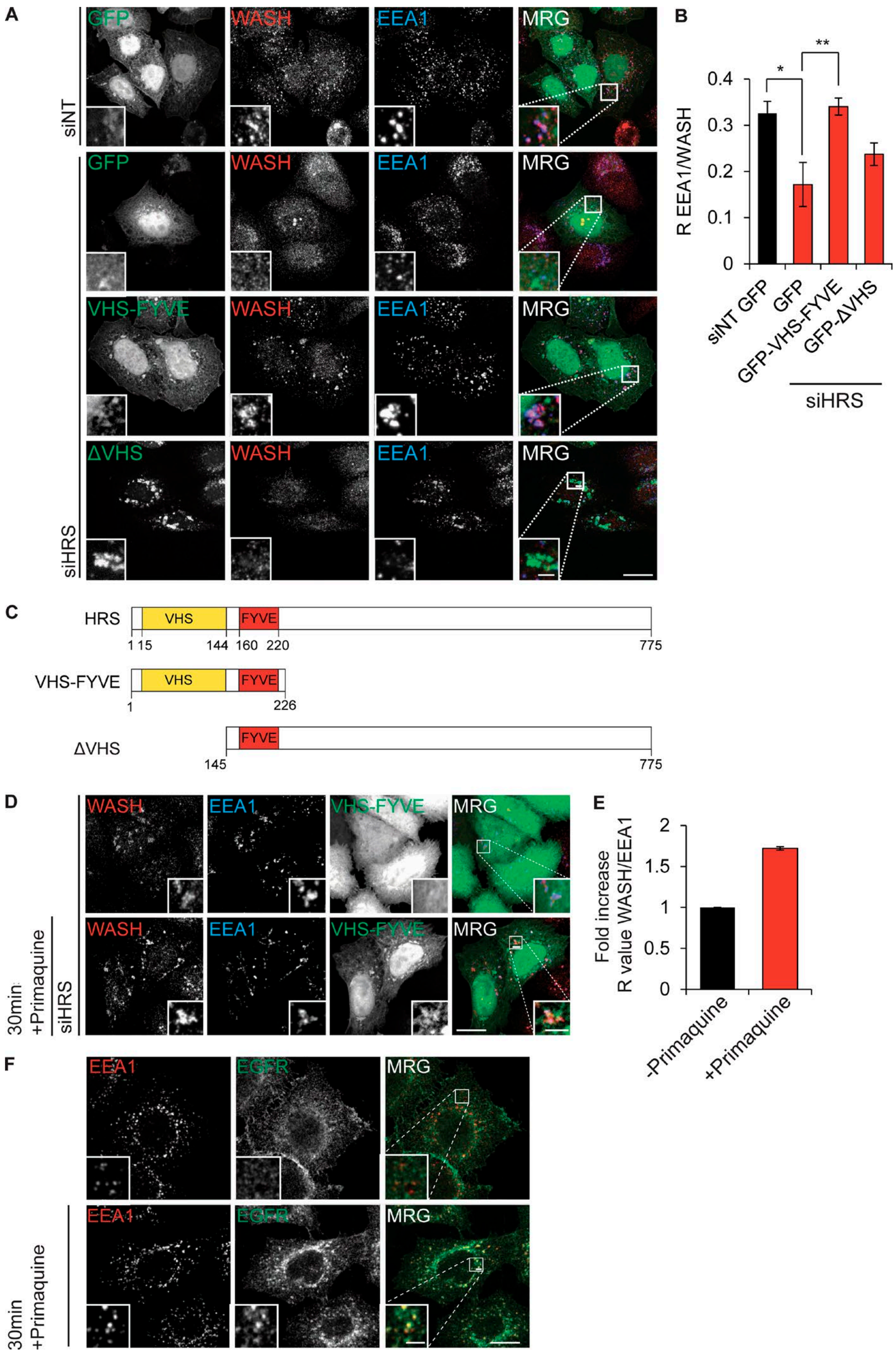

Figure S3. **VHS-FYVE domains are sufficient to recruit WASH to the endomembrane. (A and C)** HeLa cells were treated with siRNA targeting HRS for 120 h before transfection with GFP or GFP-VHS-FYVE (mHRS 1–226) domain for 24 h before fixation and antibody labeling for EEA1 and WASH. **(B)** Quantification of Pearson's R correlation value (>25 cells over two independent experiments). *, P < 0.05; **, P < 0.01. One-way ANOVA with Dunnett's post hoc test. Maximum-projection images. **(D)** HeLa cells treated as in A were pretreated with 100 µM primaquine for 30 min before fixation and labeling with the indicated antibodies. **(E)** Pearson's R correlation values divided by the siNT control (~50 cells per condition; 150 cells total). Sum intensity images. $n = 3$; error bars indicate SEM. **(F)** HeLa cells treated with 100 µM primaquine for 30 min before fixation and labeling with the indicated antibodies. Sum intensity images. Bars: (main images) 10 µm; (insets) 2 µm. MRG, merge.

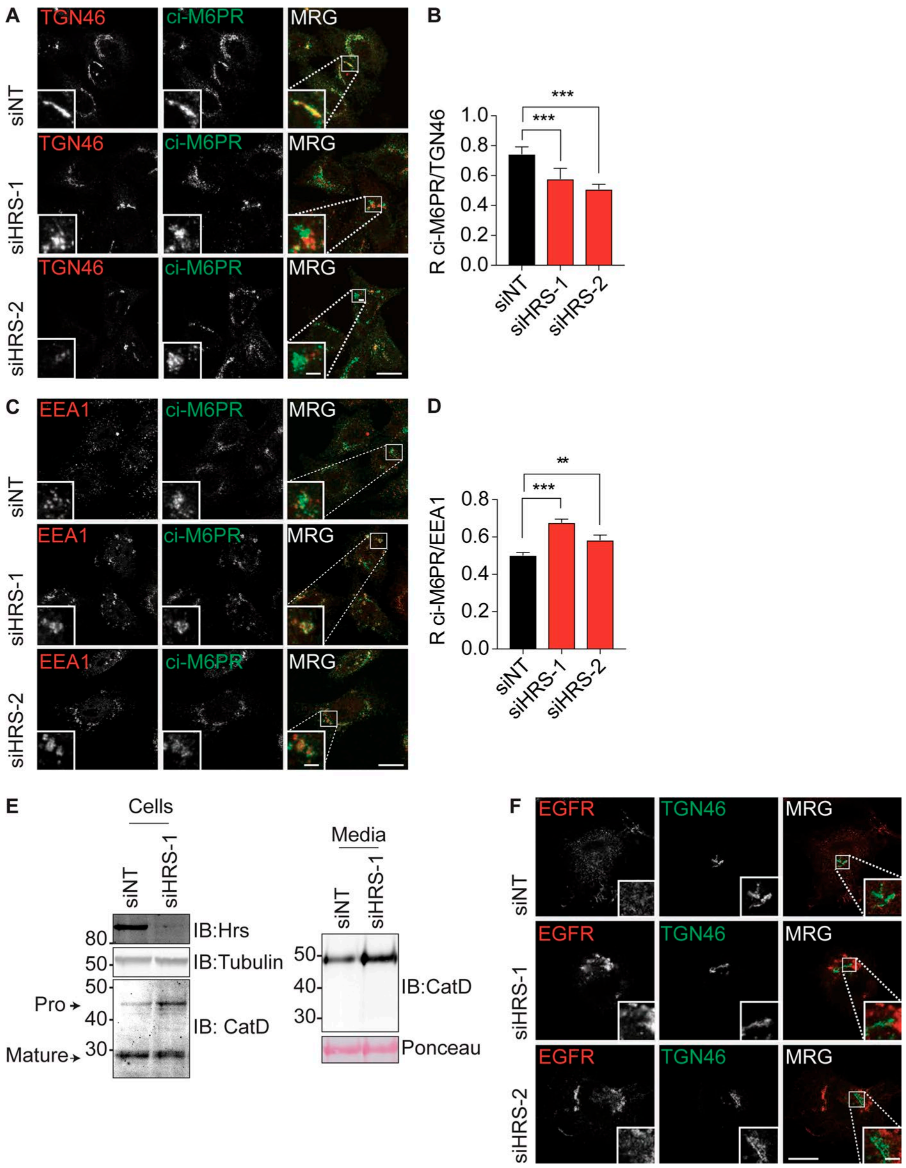

Figure S4. **HRS is required for receptor recycling. (A–D)** HeLa cells were treated with siRNA for 120 h before fixation in 4% PFA/PBS and labeling with the indicated antibodies. (Quantification of >80 cells per condition for a representative experiment; Pearson R values calculated using ImageJ; three independent experiments). Error bars indicate SEM. **(E)** TCA precipitation from the media and lysis in NP-40 buffer (representative experiment). CatD, cathepsin D; IB, immunoblot. Molecular masses are given in kilodaltons. **(F)** HeLa cells were treated with siRNA for 120 h before fixation in 4% PFA/PBS and labeling with the indicated antibodies. Bars: (main images) 10 µm; (insets) 2 µm. All images taken from a single slice. **, $P < 0.01$; ***, $P < 0.001$. Statistical analysis, one-way ANOVA for all comparisons with Dunnett's post hoc test. MRG, merge.

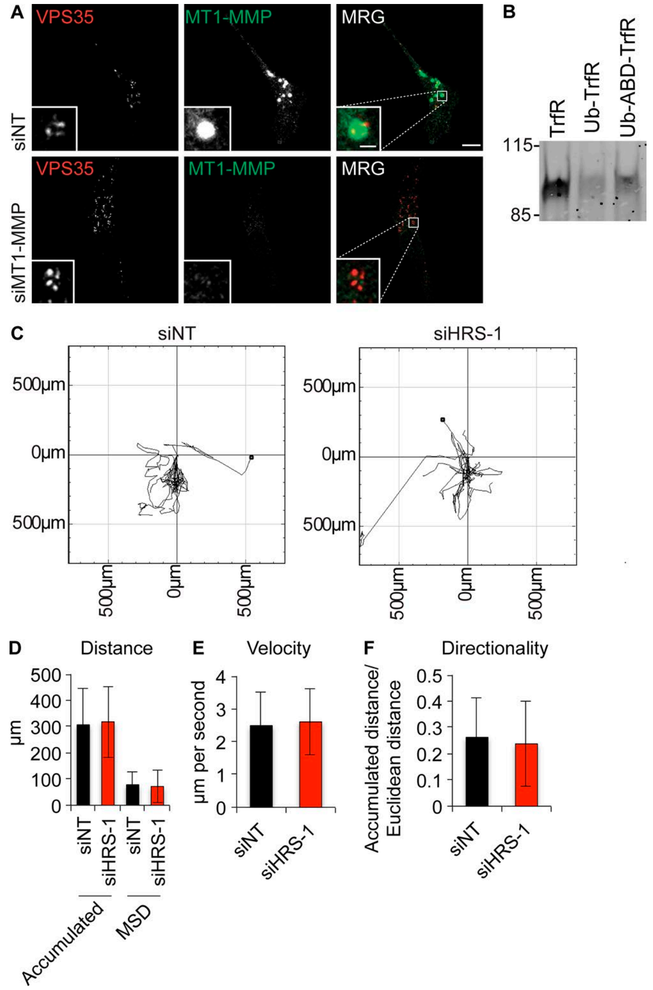

Figure S5. **HRS does not affect cell migration over a 2D substrate. (A)** MDA–MB-231 cells were treated twice with siRNA over 120 h before fixation and subsequent treatment with guanidinium hydrochloride. Images taken from a single slice. Bars: (main images) 10 µm; (insets) 2 µm. MRG, merge. **(B)** Western blot of indicated TrfR chimeras transfected into HeLa cells for 24 h and immunoprecipitated with myc tag to resolve bands on 8% SDS-PAGE gel. **(C–F)** Cells were sparsely plated on a plastic substrate and imaged for 16 h. Individual cells were tracked using manual tracker ImageJ plugin, distance (accumulated [total distance covered]), mean squared displacement (distance from start position), and directionality (accumulated distance/mean squared displacement) were measured and extracted using chemotaxis ImageJ plugin (n = 3 individual experiments; 20 cells per experiment). Error bars indicate SEM.

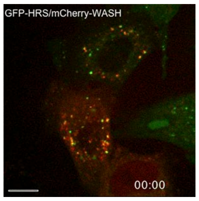

Video 1. **HeLa S3 Flp-In cells stably expressing GFP-mHRS transfected with mCherry-mWASH on a siWASH-treated background.** Bars, 10 µm. 300 ms/frame.

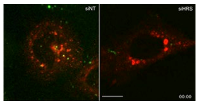

Video 2. **HeLa cells transfected with indicated siRNA (NT or HRS) for 120 h were transfected with EGFR-paGFP and mCherry-RAB4.** paGFP was activated in mCherry-positive endosomes using a pulse of 405-nm laser light. Bars, 10 µm. 600 ms/frame.

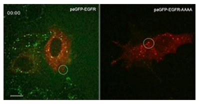

Video 3. **HeLa cells were transfected with EGFR-paGFP/EGFR-YLIP/AAAA-paGFP (actin-binding mutant) and mCherry-RAB4.** paGFP was activated in RAB4-positive endosomes using a pulse of 405-nm laser light. Bars, 10 µm. 600 ms/frame.

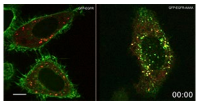

Video 4. **HeLa cells transfected with either EGFR-GFP or EGFR-YLIP/AAAA-GFP and mRFP-EEA1.** Bars, 10 µm. 600 ms/frame.

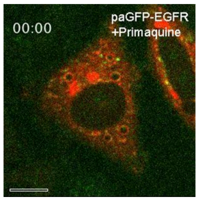

Video 5. **HeLa cells treated with indicated siRNA for 120 h were transfected with EGFR-paGFP and pCherry4-RAB4.** Cells were pretreated with 100 mM primaquine to inhibit recycling before paGFP was activated in mCherry-positive endosomes using a pulse of 405-nm laser light. Bars, 10 µm. 600 ms/frame.

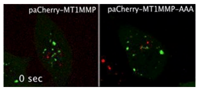

Video 6. **MDA-MB-231 cells were transfected with paCherry–MT1-MMP or paCherry–MT1-MMP–LLY/AAA (actin binding mutant) and GFP-EEA1.** paCherry was activated in EEA1-positive endosomes using a pulse of 405-nm laser light. Bars, 10 µm. 6 s/frame.
